# Sequential chemo-immunotherapy followed by standard versus reduced thoracic radiotherapy for older and/or frail stage III non-small-cell lung cancer: A randomized open-label cohort trial

**DOI:** 10.1371/journal.pmed.1005111

**Published:** 2026-05-27

**Authors:** Wei-Xiang Qi, Shuyan Li, Mengdi Wang, Huan Li, Feifei Xu, Lei Yao, Biao Yu, Linlin Chen, Gang Cai, Cheng Xu, Xianwen Sun, Zhiyao Bao, Jiayi Chen, Yi Xiang, Shengguang Zhao

**Affiliations:** 1 Department of Radiation Oncology, Ruijin Hospital, Shanghai Jiaotong University School of Medicine, Shanghai, China; 2 Shanghai Key Laboratory of Proton-therapy, Shanghai, China; 3 Department of Respiration and Critical Care Medicine, Rui Jin Hospital, Shanghai Jiao Tong University School of Medicine, Shanghai, China; Washington University in St Louis, UNITED STATES OF AMERICA

## Abstract

**Background:**

The appropriateness of concurrent chemoradiotherapy (cCRT) for older or clinically vulnerable stage III unresectable non-small-cell lung cancer (NSCLC) patients remains contentious. Furthermore, the survival implications of de-escalating thoracic radiotherapy (RT) intensity in this population have not been conclusively elucidated.

**Methods and findings:**

We conducted a phase II randomized, open-label, two-cohort (non-comparative) trial at a tertiary hospital in China (NCT05557552). Between September 30, 2022 and April 30, 2024, we enrolled 56 older and/or frail patients with stage III NSCLC who were ineligible for cCRT. The primary endpoint was the 1-year progression-free survival (PFS) rate estimated using the Kaplan–Meier method. Secondary endpoints included objective response rate (ORR), overall survival (OS), and safety. In the intention-to-treat (ITT) set, which included all 56 randomized patients who received at least one dose of study treatment, the 1-year PFS was 84.3% (95% confidence interval [CI] [70.3%, 98.3%]) in the standard RT group and 70.7% (95% CI [54.3%, 87.1%]) in the reduced RT group. In the per-protocol set (53 patients), the 1-year PFS was 82.9% (95% CI [68.9%, 98.8%]) in the standard RT group and 73.4% (95% CI [58.3%, 92.4%]), with a median follow-up of 24 months. Among 56 patients in the safety analysis set, 71.4% of patients experienced grade 3/4 adverse events (AEs) in the standard RT group and 53.6% in the reduced RT group. One patient (3.6%) in the reduced RT and three patients (10.7%) in the standardized RT experienced grade 5 AEs. The main limitations are the non-comparative design, small sample size, and lack of power to establish non-inferiority or superiority.

**Conclusion:**

The current study suggested that reduced RT combined with sequential chemo-immunotherapy might be feasible for older/frail patients intolerant to cCRT, showing numerically similar survival outcomes. These exploratory findings warrant confirmation in larger, adequately powered randomized trials.

**Trial registration:**

The trial had been registered on ClinicalTrials.gov on Sep 30, 2022.

ClinicalTrials.gov NCT05557552

## Author summary

**Why was this study done?:**

- Standard curative therapy for stage III lung cancer is often too toxic for older or frail patients.
- Effective and safer alternatives for this growing population are critically lacking.
- It was unknown whether reducing the intensity of thoracic radiotherapy within a sequential chemo‑immunotherapy regimen would compromise survival outcomes in these vulnerable patients.

**What did the researchers do and find?:**

- We conducted a phase II trial in 56 older and/or frail stage III lung cancer patients who could not tolerate standard therapy.
- We randomly assigned them to receive sequential chemo‑immunotherapy with either standard (60 Gy) or reduced‑dose (50 Gy) thoracic radiotherapy, followed by maintenance immunotherapy.
- After 24 months of follow‑up, the 1‑year progression‑free survival was comparable between groups (82.9% with standard RT and 73.4% with reduced RT), but severe toxicity was lower with reduced radiotherapy (53.6% and 71.4% experienced grade 3/4 side effects).

**What do these findings mean?:**

- For older and frail patients, a sequential treatment strategy using reduced-dose radiotherapy offers a feasible and safe option with similar short-term survival to standard radiotherapy, but this study does not demonstrate equivalence.
- These exploratory findings suggest that clinicians can balance the local control benefit of standard radiotherapy against the observed lower toxicity profile of reduced radiotherapy (53.6% and 71.4% experienced grade 3/4 side effects) for this vulnerable group.
- The results should be interpreted cautiously because of the study’s non‑comparative design and small sample size; confirmation in larger trials is needed.

## Abstract 3

In a randomized open-label trial, Wei-Xiang Qi and colleagues investigate the feasibility of dose-reduced thoracic radiotherapy to mitigate treatment related toxicities while preserving oncological outcomes in older patients with Stage III Non-small-cell Lung Cancer.

## 1. Introduction

Lung cancer remains the predominant malignancy worldwide, representing both the highest incidence among cancer types and the leading contributor to cancer-associated mortality, accounting for approximately 1.8 million annual deaths [1]. Non-small-cell lung carcinoma (NSCLC) constitutes approximately 85% of all pulmonary malignancies [2]. The landmark PACIFIC trial established a paradigm-shifting therapeutic strategy for patients with unresectable stage III NSCLC, combining concurrent chemoradiotherapy (cCRT) with subsequent 12-month durvalumab consolidation therapy, demonstrating a significant survival benefit with 5-year overall survival (OS) reaching 42.9% [3,4]. This evidence has solidified the PACIFIC regimen as the current therapeutic standard. Nevertheless, cCRT is not routinely recommended for older and/or frail patients with locally advanced NSCLC due to its high rates of toxicities [5,6]. Indeed, the incidence rate of lung cancer steeply rises among the older, and approximately 37% of the cases occur in the older who are aged >75 years [7]. In addition, frailty, a multidimensional geriatric syndrome characterized by diminished physiologic reserve, reduced resilience to stressors, and impaired adaptive capacity, is now recognized as a clinically significant determinant of outcomes in cancer patients undergoing antitumor therapy [8]. Accumulating evidence indicates that frail patients exhibit heightened susceptibility to treatment intolerance and adverse prognostic trajectories. This concern is amplified by global demographic shifts toward aging populations, with epidemiological studies reporting a doubling of frailty incidence among individuals aged ≥85 years. A recent meta-analysis of lung cancer cohorts demonstrated a pooled frailty prevalence of 45%, correlating with substantially reduced survival outcomes (hazard ratio [HR] = 3.01, 95% confidence interval [CI] [1.77, 5.10]; *p* < 0.001), underscoring its critical prognostic implications in thoracic oncology [9]. As a result, less toxic therapeutic regimens are clearly recommended to be investigated for unresectable NSCLC with older adults and/or frail.

In the GEMSTONE-301 trial, both sequential and cCRT followed by immune checkpoint inhibitors (ICIs) were evaluated [10], and sub-group analysis indicated that both sequential and cCRT showed comparable efficacy [HR: 0.59 and 0.66], which suggested that the use of sequential chemoradiotherapy (SCRT) followed by ICIs could be an optimal option for patients with locally advanced, unresectable, stage III NSCLC who cannot tolerate cCRT. Additionally, a subgroup analysis of the PACIFIC study revealed that a certain group of patients received less than 60 Gy of radiotherapy [4]. This patient cohort exhibited adverse prognostic factors including elevated tumor burden and suboptimal radiation dosing (<60 Gy). Notably, ICIs maintenance therapy demonstrated sustained clinical benefits in both progression-free survival (PFS) and OS, with enhanced survival advantage observed specifically in patients receiving reduced radiotherapy (RT) regimens. To address this therapeutic dichotomy, we designed this study to prospectively evaluated the efficacy-safety profile of SCRT followed by ICIs consolidation in older and/or frail patients with stage III NSCLC. Furthermore, we investigated the feasibility of dose-reduced thoracic radiotherapy to mitigate treatment-related toxicities while preserving oncological outcomes. We hypothesized that reduced-dose radiotherapy would offer comparable PFS with lower toxicity.

## 2. Methods

### 2.1 Ethics statement

The study protocol and all amendments were reviewed and approved by the Institutional Review Board of Ruijin Hospital, Shanghai Jiao Tong University School of Medicine (approval No. 2021-189). The study was conducted in accordance with the Declaration of Helsinki and Good Clinical Practice. Written informed consent was obtained from all patients prior to any study-related procedures.

### 2.2 Study design and patients

Our trial was an open, single-center, randomized, two-cohort prospective clinical study conducted at Ruijin Hospital, Shanghai Jiao Tong University School of Medicine in China. It was designed as an exploratory study to evaluate the feasibility and preliminary efficacy of two sequential treatment approaches, rather than a confirmatory non-inferiority or superiority trial. The Trial Protocol is provided in S1 Protocol and S2 Protocol. An independent Data Safety Monitoring Board (DSMB) was established to monitor patient safety and study conduct; no early stopping rules were triggered. This study is reported according to the CONSORT 2025 guideline (S1 CONSORT Checklist). This trial adhered to the CONSERVE‑CONSORT guideline for reporting modifications due to extenuating circumstances (S1 CONSERVE‑CONSORT Checklist). We chose a two-cohort, non-comparative design because no prospective data existed for this frail population, and the primary aim was to estimate the feasibility and preliminary efficacy of each radiotherapy dose separately, rather than to test superiority or non-inferiority.

**Fig 1:**
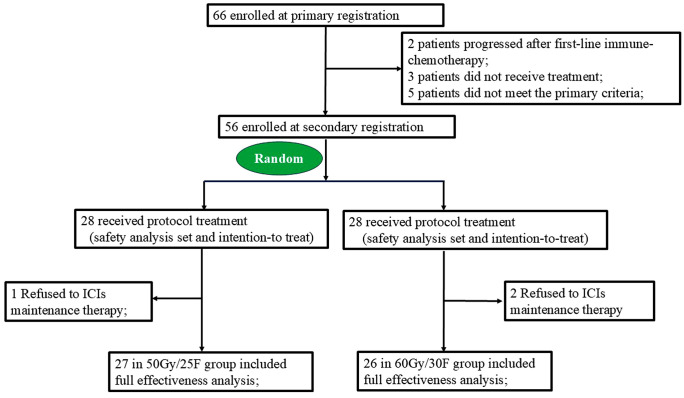
Patient flowchart. Abbreviations: ICIs, immune checkpoint inhibitors.

This study had 2 stages of registration (Fig 1). We used the following main inclusion criteria for the primary registration: (1) 18 years or older at time of study entry; (2) Histologically documented diagnosis of unresectable stage III NSCLC; Note: For non-squamous cell carcinoma: participant with known positive epidermal growth factor receptor (EGFR) sensitive mutation must be excluded; (3) Fully-informed written consent obtained from patients; (4) Unfit for cCRT as determined by the multi-disciplinary team board due to one of the following reasons: [1] Eastern Cooperative Oncology Group (ECOG) Performance Status (PS) of 2; [2] Age ≥ 70 with ECOG PS 0–1; [3] Age ≥ 65 with Charlson Comorbidity Index (CCI) = 1. Thus, “older” in this study is explicitly defined as age ≥ 70 years. For patients who did not meet this age threshold, eligibility required the presence of significant comorbidities (CCI ≥ 4) to signify increased vulnerability. It should be noted that this pragmatic definition of frailty/unfitness, based on age, ECOG PS, and CCI, was employed for feasibility. We acknowledge its limitations compared to a comprehensive geriatric assessment; (5) Adequate bone marrow, liver and kidney function; (6) Life expectancy of at least 3 months; (7) At least one measurable [Response Evaluation Criteria in Solid Tumors (RECIST) 1.1], thoracic lesion that can be irradiated; (8) Histologic or cytologic confirmation of NSCLC; (9) Adequate pulmonary function with forced expiratory volume in 1 second (FEV1) >1 L or >30% of predicted value and diffusing capacity of the lungs for carbon monoxide (DLCO) >30% of predicted value. The major exclusion criteria were for the primary registration were as follows: (1) Previous chemo-, immuno- or radiotherapy for NSCLC; (2) Major surgical procedure last 28 days; (3) History of allogenic organ transplantation, autoimmune disease, immunodeficiency, hepatitis or human immunodeficiency virus (HIV); (4) Uncontrolled intercurrent illness; (5) Other active malignancy; (6) Leptomeningeal carcinomatosis; (7) Immunosuppressive medication; (8) Pregnant or breastfeeding women.

The key eligibility criteria for the secondary registration included the following: (1) The treatment response was evaluated as complete response, partial response or stable disease after 4–6 cycles of chemo-immunotherapy according to RECIST 1.1. The range of 4–6 cycles allowed for response-adapted treatment duration based on individual tolerance and tumor response, reflecting real-world practice in this frail population; (2) agreed to received thoracic radiotherapy and ICIs maintenance therapy; The full eligibility criteria were listed in the protocol. The primary efficacy analysis was performed on the intention-to-treat (ITT) set, which included all randomized patients according to their original allocation, consistent with the ITT principle. We documented AEs according to the National Cancer Institute Common Terminology Criteria for Adverse Events, version 4.03. Tumor response evaluation was performed following the RECIST 1.1. Follow-up was conducted every 2 months.

### 2.3 Outcomes

The primary endpoint of the study was the 1-year PFS rate. Secondary end points included objective response rate (ORR), OS, and safety. PFS was defined as the time from the initiation of study treatment to any disease progression. ORR was defined as the proportion of patients with a confirmed complete or partial response. OS was defined as the time from the initiation of the study treatment to any-cause death or last follow-up, whichever occurred first.

### 2.4 Analysis sets

The following analysis sets were defined: ITT set (formerly Full Analysis Set, FAS): Following the ITT principle, we included all randomized patients (*n* = 56), all of whom received at least one dose of study treatment (induction chemo-immunotherapy, radiotherapy, or ICIs). We analyzed these patients according to the group to which they were originally assigned. No patients were excluded from the primary efficacy analysis. Per-Protocol Set (PPS): A subset of the FAS, including patients who completed the induction chemo-immunotherapy, received the full course of assigned radiotherapy (with concurrent ICIs), and received at least one cycle of ICIs maintenance therapy, without major protocol violations that could affect efficacy evaluation. Safety Set (SS): Included all patients who received at least one component of the study treatment (induction therapy, radiotherapy, or ICIs) after the first registration. We performed safety analyses according to the treatment actually received.

### 2.5 Handling of missing data

We did not impute missing data for any analysis. The approach to missing data was determined by the type of variable and analysis. For time-to-event endpoints (PFS, OS): Participants who were lost to follow-up or who had not experienced the event (progression or death) by the data cut-off date were censored at the date of their last known clinical or radiographic assessment. For baseline characteristics and safety analyses: No imputation was performed for missing baseline or safety data. All analyses were based on available data.

### 2.6 Study procedures

We administered ICIs intravenously over ≥60 min on Day 1 of each 21-day cycle. ICIs were administered firstly, and chemotherapy was started 30 min after the completion of ICIs, if they were given on the same day. Acceptable chemotherapy included Etoposide/Vinorelbine/Paclitaxel/Docetaxel/Pemetrexed + platinum for 4–6 cycles, with the exclusion of Gemcitabine. The range of 4–6 cycles for the sequential chemotherapy component was designed to align with standard curative-intent treatment duration while allowing for individualized adaptation based on tolerance and response in this frail population. The range of 4–6 cycles for the sequential chemotherapy component was designed to align with standard curative-intent treatment duration for locally advanced NSCLC, recognizing that some frail patients may require fewer cycles. This duration differs from the 2–3 cycles used in PACIFIC-6 [11] and GEMSTONE-301 [12], where patients received SCRT (chemotherapy followed by radiotherapy without concurrent immunotherapy during the induction phase). In our trial, the induction phase consists of 4–6 cycles of platinum-doublet chemotherapy plus an ICI, which is consistent with standard first-line treatment for advanced NSCLC as recommended by the European Society for Medical Oncology (ESMO) Clinical Practice Guideline [13] and the National Comprehensive Cancer Network (NCCN) guidelines (version 4.2026) [14] for locally advanced disease when given as part of definitive chemoradiotherapy or sequential therapy. The range allows for individualized adaptation based on tolerance and response in this frail population.

After receiving 4–6 cycles of induction chemo-immunotherapy, frail/older stage III Patients with NSCLC were randomized in a 1:1 ratio by an independent study coordinator (Biao Yu) using a computer-generated simple randomization sequence to receive ICIs combined with either standard thoracic radiotherapy dose 60 Gy/30Fx (standard group cohort A) or reduced thoracic radiotherapy dose 50 Gy/25Fx (reduced group cohort B). The reduced radiotherapy dose of 50 Gy in 25 fractions was selected based on the following considerations: (1) it represents a biologically effective dose [biologically effective dose for an α/β ratio of 10 Gy (BED10) ≈ 60 Gy] that was commonly used in palliative thoracic radiotherapy for locally advanced NSCLC while being associated with lower rates of grade ≥3 radiation pneumonitis and esophagitis compared to 60 Gy; (2) data from the PACIFIC trial subgroup analysis suggested that patients receiving <60 Gy still derived benefit from consolidation immunotherapy; (3) the reduced dose was intended to mitigate the increased toxicity risk when combining concurrent ICIs with thoracic radiotherapy in this frail population, particularly the risk of pneumonitis.

The investigator who enrolled participants was unaware of the upcoming allocation. The random allocation sequence was securely stored and was only accessible to the independent study coordinator, who assigned the enrolled participants to their groups according to the sequence. After a patient’s eligibility for radiotherapy was confirmed (second registration), the coordinator assigned the patient to either the standard or reduced RT group according to the next entry on the sequence. The clinicians enrolling patients had no access to the allocation sequence. Patients then received ICIs combined with either standard or reduced radiotherapy dose. And concurrent ICIs were administered during radiotherapy every 21 days. The rationale for combining immunotherapy concurrently with radiotherapy was to potentially enhance local tumor control during the radiation phase, with the reduced RT dose (50 Gy) selected to mitigate the risk of increased toxicity, particularly pneumonitis, in this frail cohort. After thoracic RT, we continued ICI maintenance for at least 6 months (8 cycles) or until progression, unacceptable toxicity, or death.

### 2.7 Statistical analysis

We used Simon’s two-stage method to design this study. Assuming an improvement in the 1-year PFS rate from 20% [15] to 40% after receiving sequential chemo-immunotherapy followed by thoracic radiotherapy in patients with stage III NSCLC who were unable to tolerate cCRT, enrolled patients were randomized into cohort A and cohort B, with each cohort of 25 patients. The historical control rate of 20% was derived from the Japan Clinical Oncology Group (JCOG) 0301 trial, which evaluated radiotherapy alone or with low-dose chemotherapy in older patients with NSCLC, representing a relevant benchmark for our frail population [11]. After accounting for a 10% patient dropout rate, a total of 56 patients (28 in each cohort) were required to be enrolled. Continuous data were reported as median (interquartile range [IQR] or range), and categorical data were reported as frequency (percentage). We estimated survival using the Kaplan–Meier method and computed 95% CIs with the Brookmeyer-Crowley method. Between-group comparisons for time-to-event endpoints (PFS, OS) were performed using the log-rank test. HR with 95% CIs were estimated using Cox proportional hazards models when appropriate. Comparisons of categorical variables, including ORR and toxicity rates, were performed using the Chi-squared test or Fisher’s exact test as applicable. All *p*-values are presented as exploratory and descriptive, given the study’s non-comparative design and sample size. No adjustments were made for multiple comparisons. The ITT set served as the primary analysis population for efficacy outcomes. The per-protocol set was used for sensitivity analyses.

## 3. Results

### 3.1 Baseline characteristics

From September 2022 through April 2024, we screened 66 patients for eligibility and enrolled 56. We enrolled the first patient on September 30, 2022, and the last patient on April 20, 2024. The enrollment period was extended from the planned 12 months to 18 months due to the Coronavirus Disease (COVID)-19 pandemic, which temporarily reduced patient referrals. Although the trial was registered on the same day as first patient enrollment (September 30, 2022), the protocol and registration materials had been finalized prior to initiation. The delay was due to administrative processing at the institutional level; no patients were enrolled before registration was complete. We confirm that no related or similar trials are being conducted by our group without prospective registration. Any future trials will be registered before first patient enrollment. A flow diagram is shown in Fig 1. The baseline characteristics were listed in Table 1. Participants had a median (range) age of 68 (65−79) years; 50 (89.3%) were men. Seventy-three% of patients had squamous cell carcinoma, with 19.6% of adenocarcinoma. A total of 23.2% presented with >50% programmed cell death ligand 1 (PD-L1) expression, 33.9% with 1%–50% and 16.1% with 0%. As for the comorbidities, the most commonly disease was hypertension (37.5%), followed by chronic obstructive pulmonary disease (COPD) (32.1%), diabetes (25%) and cerebrovascular disease (21.4%). As for the induction chemotherapy, 75% of patients treated with nab-paclitaxel combined with carboplation, and 16.1% patients treated with pemetrexed with carboplatin. In addition, 89.3% treated with anti-programmed cell death protein 1 (anti-PD-1), (pembrolizumab, nivolumab, sintilimab, toripalimab, camrelizumab, or tislelizumab) and 10.7% with anti-PD-L1 inhibitors (durvalumab, atezolizumab, or sugemalimab). Two patients in standardized RT and one patient in reduced RT group refused to receive ICIs maintenance therapy. Therefore, the per protocol set was 26 in standardized RT and 27 in reduced RT group, respectively.

**Table 1:** Baseline characteristics of included patients.

	Overall	Reduced RT	Standard RT
**Sex**			
**Male, *n***	50(89.3%)	24(85.7%)	26(92.9%)
**Female, *n***	6(10.7%)	4(14.3%)	2(7.1%)
**Age**			
**Median, range, years**	68(65–79)	67.5(65–79)	68.5(65–79)
**≥70**	22(39.3%)	9(32.1%)	13(46.4%)
**<70, ≥65**	34(60.7%)	19(67.9%)	15(53.6%)
**Histologic types**			
**SCC**	41(73.2%)	18(64.3%)	13(46.4%)
**adenocarcinoma**	11(19.6%)	8(28.6%)	3(10.7%)
**Not specified**	4(7.1%)	2(7.1%)	2(7.1%)
**PD-L1 expression**			
**0%**	9(16.1%)	5(17.9%)	4(14.3%)
**1%–50%**	19(33.9%)	10(35.7%)	9(32.1%)
**>50%**	13(23.2%)	7(25%)	6(21.4%)
**Not done**	15(26.8%)	5(17.9%)	10(35.7%)
**ECOG status**			
**0**	10(17.9%)	6(21.4%)	4(14.3%)
**1**	32(57.1%)	18(64.3%)	14(50.0%)
**2**	14(25.0%)	4(14.3%)	10(35.7%)
**Comorbidity**			
**Hypertension**	21(37.5%)	9 (32.1%)	12(57.1%)
**Diabetes**	14(25%)	8(28.6%)	6(21.4%)
**COPD**	18(32.1%)	9(32.1%)	9(32.1%)
**Ischemic heart disease**	6(10.7%)	6(21.4%)	0
**Arrhythmia**	3(5.4%)	2(7.1%)	1(3.6%)
**Cerebrovascular disease**	12(21.4%)	7(25%)	5(17.9%)
**Renal insufficiency**	3(5.4%)	1(3.6%)	2(7.1%)
**Nephrotic syndrome**	1(1.8%)	1(3.6%)	0
**gastric ulcer**	2(3.6%)	2(7.1%)	0
**Charlson comorbidity index,** **CCI**			
**4–5**	37(66.1%)	18(64.3%)	19(67.9%)
**6–9**	19(33.9%)	10(35.7%)	9(32.1%)
**Induction chemotherapy regimens**			
**Nab-paclitaxel +carboplatin**	42 (75%)	17(60.7%)	25(89.3%)
**Paclitaxel+ carboplatin**	5(8.9%)	3(10.7%)	2(7.1%)
**Pemetrexed + carboplatin**	9(16.1%)	8(28.6%)	1(3.6%)
**Types of PD-1/PD-L1**			
**Anti-PD-1**	50(89.3%)	25(89.3%)	25(89.3%)
**Anti-PD-L1**	6(10.7%)	3 (10.7%)	3(10.7%)
**Cycles of induction chemoimmunotherapy**			
**4 cycles**	38(67.9%)	19(67.9%)	19(67.9%)
**5 cycles**	1(1.8%)	0	1(3.6%)
**6 cycles**	17(30.4%)	9(32.1%)	8(28.6%)

Abbreviations: RT, radiotherapy; COPD, chronic obstructive pulmonary disease; SCC, squamous cell carcinoma.

### 3.2 Efficacy

**Fig 2:**
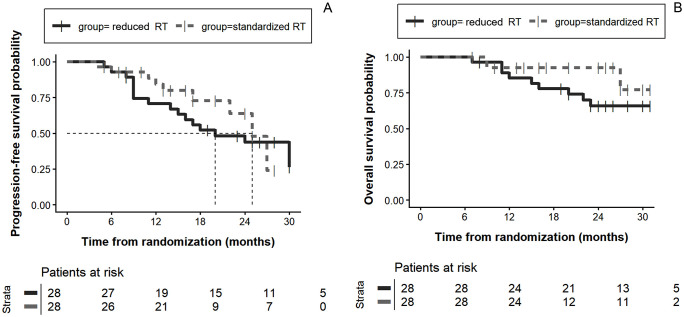
Kaplan–Meier curves for progression-free survival (PFS) and overall survival (OS) in the intention-to-treat (ITT) set. Abbreviations: RT, radiotherapy; ITT, intention-to-treat. Note: Survival curves were estimated using the Kaplan–Meier method. Between-group comparisons were descriptive only due to the non-comparative design.

As of April 30, 2025, with a median follow-up of 24 months (95% CI [23, 26]), no patients were lost to follow-up in either group. For the ITT set (56 patients), the 1-year PFS was 84.3% (95% CI [70.3%, 98.3%]) in the standard RT group versus 70.7% (95% CI [54.3%, 87.1%]) in the reduced RT group (Fig 2A). And The 1-year OS in the ITT set was comparable between the two groups [92.6% (95% CI [82.2%, 100.0%]) for standard thoracic RT group and 85.3% (95% CI [73.0%, 99.7%]) for reduced thoracic RT group, Fig 2B]. The median PFS was 20 months for reduced group and 25 months for standardized group.

**Fig 3:**
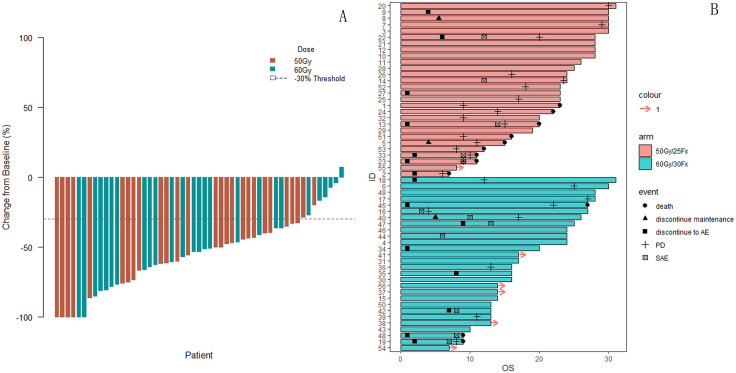
Waterfall Plot in the per protocol set and Swimmer Plot in the full analysis set for Individual Patients. Abbreviations: PD, progression disease; SAE, severe adverse event; AE, adverse event.

For the per protocol set, the 1-year PFS in standard thoracic RT group (60 Gy/30Fx) and reduced thoracic RT group (50 Gy/25Fx) was 82.9% (95% CI [68.9%, 98.8%]) and 73.4% (95% CI [58.3%, 92.4%], S1A Fig). And the 1-year OS was also comparable between the two groups 92.0% (95% CI [82.0%, 100.0%]) for standard thoracic RT group and 84.7% (95% CI [72.0%, 99.7%]) for reduced thoracic RT group, S1B Fig). The median PFS was 20 months for reduced group and 27 months for standardized group. The median OS was not reached in the two groups. For the reduced RT group, the best ORR was 92.6% (25 of 27), including 4 patients (14.8%) with a confirmed complete response and 21 patients (77.8%) with a confirmed partial response (Fig 3A). For the standardized RT group, the best ORR was 92.3% (24 of 26), including 2 patients (7.7%) with a confirmed complete response and 22 patients (84.6%, Fig 3B) with a confirmed partial response. A total of nine patients (33.3%) in the reduced RT group and three patients (11.5%) in the standardized RT group died by the end of follow-up, and the Median OS was not reached in the two groups.

### 3.3 Treatment duration

All 56 patients completed induction chemo-immunotherapy and planned thoracic radiotherapy, and 53 received at least 1 cycle of ICIs maintenance therapy. The median ICIs maintenance duration was 6 months (95% CI [4, 12]). Of them, 32 patients (57.1%) complete more than 6 months ICIs maintenance and 21 patients (37.5%) completed 1-year ICIs maintenance. A total of 14 patients (50%) in both groups experienced interruption of ICIs maintenance due to treatment toxicities, mainly because of pneumonitis including radiation pneumonitis (75%), and 7 patients in reduced thoracic RT group and 6 patients in standardized thoracic RT group resumption of immunotherapy.

As of April 30, 2025, 6 patients (10.7%) were still receiving ICIs maintenance without progression. A total of 26 patients experienced disease progression. For the reduced thoracic RT group, 11 patients experienced local-region progression after thoracic RT, three with distant metastasis and three patients developed malignant pleural effusion. In the standard RT group, while five patients developed local regional progression, one patient presented intrapulmonary and distant metastasis, and three experienced distant metastases. More patients in reduced thoracic RT developed local-regional progression in comparison with standardized RT group (39.3% and 21.4%).

### 3.4 Safety

Treatment-related adverse events (TRAEs) are summarized in Table 2. The most common grade ≥3 TRAEs were -hematologic- toxicities, occurring in 12 patients (42.9%) in the reduced RT group and 14 patients (50.0%) in the standard RT group. Among these, neutropenia (grade 3: 14.3% versus 10.7%; grade 4: 7.1% versus 3.6%), leukopenia (grade 3: 14.3% versus 21.4%), and thrombocytopenia (grade 4: 3.6% versus 7.1%) were the most frequent events. Non-hematologic grade 3/4 TRAEs were observed in 3 patients (10.7%) in the reduced RT group and 6 patients (21.4%) in the standard RT group; these included pneumonitis, lung infection, dermatitis, and atrial fibrillation.

**Table 2:** Treatment-related toxicity.

Toxicities	Chemoimmunotherapy followed by reduced RT of 50 Gy/25Fx (*n* = 28)				Chemoimmunotherapy followed by standard RT of 60 Gy/30Fx (*n* = 28)			
	Grade 1/2	Grade 3	Grade 4	Grade 5	Grade 1/2	Grade 3	Grade 4	Grade 5
Hematologic toxicities								
Anemia	19(67.9%)				25(89.3%)	1(3.6%)		
Leukopenia	11(39.3%)	4(14.3%)	1(3.6%)		11(39.3%)	6(21.4%)		
Neutropenia	6(21.4%)	4(14.3%)	2(7.1%)		10(21.4%)	3(10.7%)	1(3.6%)	
Thrombocytopenia	8(28.6%)		1(3.6%)		8(28.6%)	1(3.6%)	2(7.1%)	
Increased ALT	2(7.1%)				4(14.3%)			
Increased AST	2(7.1%)				3(10.7%)			
Non-hematologic toxicities								
Pneumonitis	7(25%)	1(3.6%)		1(3.6%)	7(25%)	3(10.7%)		
Lung infection	5(17.9%)	1(3.6%)			4(14.3%)	1(3.6%)		
Dermatitis		1(3.6%)				1(3.6%)		
Atrial fibrillation						1(3.6%)		
Hemorrhage								2(7.1%)
pancreatitis	1(3.6%)							
Myocarditis	1(3.6%)				1(3.6%)			
Hypothyroidism	1(3.6%)				1(3.6%)			
Myocardial infarction								1(3.6%)
Overall		11(39.3%)	4(14.3%)	1(3.6%)		17(60.7%)	3(10.7%)	3(10.7%)

Abbreviations: RT, radiotherapy.

Regarding pneumonitis (including radiation pneumonitis), any-grade events occurred in 8 patients (28.6%) in the reduced RT group (grade 1/2: 7 [25.0%]; grade 3: 1 [3.6%]; grade 5: 1 [3.6%]) and 10 patients (35.7%) in the standard RT group (grade 1/2: 7 [25.0%]; grade 3: 3 [10.7%]; no grade 4). The single grade 5 pneumonitis event in the reduced RT group occurred 7 months after thoracic radiotherapy; the patient had underlying chronic obstructive pulmonary disease and developed progressive dyspnea unresponsive to high-dose corticosteroids. No grade 5 pneumonitis occurred in the standard RT group.

Fatal treatment‑related events were reported in one patient (3.6%) in the reduced RT cohort (pneumonitis) and three patients (10.7%) in the standard RT cohort: two died from hemorrhage (one pulmonary hemorrhage, one gastrointestinal hemorrhage) and one from myocardial infarction. All fatal events were considered possibly or likely related to treatment by the investigator and the Data Safety Monitoring Board.

The overall incidence of grade ≥3 TRAEs (any type) was lower in the reduced RT cohort than in the standard RT cohort (57.1% [16/28] versus 82.1% [23/28]), primarily driven by differences in hematologic and pulmonary toxicities. No treatment‑related deaths were attributed to immunotherapy alone.

## 4. Discussion

For patients with unresectable, stage III NSCLC who were ineligible for cCRT—a growing demographic often characterized by advanced age, frailty, or significant comorbidities—identifying effective yet tolerable curative-intent strategies remained a paramount challenge. Our phase II randomized trial directly addressed this unmet need. The primary finding was that a sequential approach of chemo-immunotherapy followed by thoracic radiotherapy and ICI maintenance appeared feasible and showed promising activity in this vulnerable population. More specifically, reducing the radiotherapy dose from a standard 60 to 50 Gy appeared to offer a meaningful trade-off: it yielded comparable survival outcomes while appearing to reduce severe toxicity, particularly fatal events. Our results do not demonstrate equivalence; rather, they show that reduced-dose RT was not statistically significantly inferior to standard RT in this exploratory analysis, but the study was not powered for non-inferiority. Therefore, we cannot conclude equivalence.

To contextualize these results, it was helpful to view current treatment options for “cCRT-unfit” patients as a spectrum of intensity and efficacy. At one end are regimens for patients deemed unfit for any chemotherapy, such as radiotherapy alone followed by consolidation immunotherapy (SPIRAL-RT) [16] or durvalumab after radiotherapy (DUART) [17]. These strategies prioritize tolerability, with reported median PFS in the range of 9−11 months. At the other end of the spectrum is the PACIFIC-6 paradigm [18], which applies standard SCRT followed by durvalumab consolidation in a similarly defined “unfit” population, reporting a 12-month PFS of 49.6% with a favorable safety profile. Our study investigates an intermediate-intensity approach: sequential chemo-immunotherapy followed by radiotherapy concurrently with ICIs. This design was conceptually aligned with the DOLPHIN trial but incorporates prior systemic therapy. Our observed 1-year PFS rates (73.4%−82.9%) were numerically higher than those in PACIFIC-6 and DOLPHIN (72.1%) [19], while the toxicity profile was more pronounced than in PACIFIC-6 but appeared mitigated compared to historical expectations for concurrent radio-immunotherapy. This positions our reduced-dose RT regimen (50 Gy) as a potentially viable option on this spectrum, offering a balance between the robust efficacy of combined modality therapy and the imperative for reduced toxicity in a frail cohort.

The recent DEDALUS trial [20] investigated a similar strategy of induction chemo-immunotherapy followed by hypo-fractionated radiotherapy (45 Gy in 15 fractions) concurrent with durvalumab. This regimen reported a promisingly low incidence of grade 3/4 treatment-related AEs (28.0%), with notably zero severe toxicities attributed to radiotherapy. In contrast, our study employed conventional fractionation (50 Gy/25Fx or 60 Gy/30Fx) and observed higher overall rates of severe toxicity. This difference may be partly attributable to the distinct toxicity profiles and potentially lower biologically effective dose of the hypo-fractionated regimen used in DEDALUS. However, it is noteworthy that in DEDALUS, 28% of patients (7 of 25) did not proceed to radiotherapy following induction, primarily due to disease progression. In our cohort, all 56 patients successfully completed induction and proceeded to planned radiotherapy, suggesting potential differences in the efficacy of the induction regimen or in patient selection. Together, these studies highlight that within the sequential chemo-immunotherapy backbone, both radiotherapy dose (as tested in our trial) and fractionation schedule (as in DEDALUS) are critical modifiable factors that significantly influence the efficacy-toxicity balance. The optimal combination for frail patients warrants further investigation in comparative studies.

Regarding recurrence patterns, local-regional progression emerged as the predominant mode of treatment failure in our cohort, aligning with findings from the PACIFIC Trial [4,21]. In this analysis, local-regional progression occurred in 39.3% of patients receiving reduced thoracic RT, compared to 21.4% in the standardized RT group. This numerical difference, though not statistically significant, suggested that lower RT dose might be associated with less effective local control, a trade-off that must be balanced against toxicity reduction.

Regarding safety, four patients experienced grade 5 (fatal) treatment-related toxicities: one (3.6%) in the reduced thoracic RT group and three (10.7%) in the standardized RT group. The treatment-related mortality rate in the reduced RT cohort (3.6%) aligned with the PACIFIC trial (4.4%), whereas the standardized RT group exhibited higher mortality [4]. For grade 3/4 toxicities, 71.4% of patients in the standardized RT group experienced severe adverse events (AEs), compared to 53.6% in the reduced RT group. These rates were consistent with the DOLPHIN trial (52.9%) [19], but exceed the PACIFIC trial’s reported 29.9% [4]. The most frequent grade 3/4 hematologic toxicities were leukopenia and neutropenia, potentially attributable to reduced tolerance to sequential chemoimmunotherapy and thoracic RT among older and/or frail patients with NSCLC. Common non-hematologic grade 3/4 AEs included pneumonitis and lung infection. The incidence of grade ≥3 pneumonitis (including radiation pneumonitis) (7.6% in reduced RT and 10.7% in standardized RT) surpassed rates in the PACIFIC trial (3.4%) but paralleled the DOLPHIN trial (11.8%). This discrepancy might reflect the PACIFIC trial’s exclusion of patients with preexisting grade ≥2 pneumonitis post-chemoradiation, whereas the DOLPHIN and current trial permitted concurrent ICI administration. The rationale for combining immunotherapy concurrently with radiotherapy in this frail population was to potentially enhance local tumor control during the radiation phase through immune-mediated effects, such as immunogenic cell death and reversal of local immunosuppression. However, this approach carries an increased risk of pneumonitis, which led us to evaluate a reduced radiotherapy dose (50 Gy) as a strategy to mitigate toxicity while preserving efficacy. Our results suggest that this trade-off may be acceptable, but the optimal concurrent regimen requires further study.

All 56 patients successfully completed induction chemo-immunotherapy and the planned thoracic radiotherapy. However, in the PACIFIC trial, 8% of Patients with NSCLC receiving cCRT did not finish the prescribed RT (60 Gy) [21]. Given that intrapulmonary progression was the primary cause of treatment failure after thoracic RT, ensuring the completion of thoracic RT was crucial for enhancing local control in patients with unresectable, locally advanced NSCLC. Furthermore, the median duration of ICIs maintenance therapy in the current study was 6 months. Of the patients, 32 (57.1%) completed more than 6 months of maintenance, and 21 (37.5%) completed 1 year of ICIs maintenance. While 50% of the patients experienced interruptions in ICIs maintenance, primarily due to pneumonitis (including radiation pneumonitis), 7 patients (50%) in the reduced thoracic RT group and 6 patients (42.9%) in the standardized thoracic RT group resumed immunotherapy. The high completion rates of thoracic RT and ICIs maintenance in this study suggested that the sequential chemo-immunotherapy regimen combined with thoracic RT followed by ICIs was a tolerable treatment approach for older and/or frail patients with NSCLC. Interestingly, despite the frail population, toxicity during the induction chemo-immunotherapy phase was manageable, with only a small proportion not proceeding to radiotherapy. This might be attributed to careful patient selection, dose modifications, and supportive care.

The trial had the following limitations. Firstly, our study was a randomized cohort trial and lacked direct comparisons. We designed it as an exploratory study; therefore, we present between-group comparisons as descriptive only, and they lack power to establish non-inferiority or superiority. Consequently, we cannot conclude that reduced RT is comparable to standard RT, and future randomized studies must directly compare these two regimens. Secondly, we assessed frailty using the CCI, a simple tool for evaluating patient vulnerability [22]. However, the CCI and age alone might not accurately identify frail patients. Further efforts by oncology professionals were required to establish standardized and accurate methods for identifying frail patients and tailoring treatment accordingly [8]. Thirdly, we included both Programmed Cell Death-1 (PD-1) and Programmed Cell Death Ligand 1 (PD-L1) inhibitors, which may complicate interpretation of the results. However, durvalumab [4] and sugemalimab [12] had been approved for consolidation therapy in the treatment of locally advanced NSCLC, and several trials were currently investigating the role of ICIs maintenance therapy in NSCLC. Therefore, the wide usage of ICIs reflected current clinical practice. Fourthly, PD-L1 expression data were missing for a subset of patients, limiting biomarker analysis. Finally, the sample size was relatively small, and the follow-up duration might not be sufficient to capture long-term outcomes and toxicities.

In conclusion, we demonstrated that sequential chemo‑immunotherapy plus thoracic RT followed by ICIs maintenance might be effective and tolerable for older/frail stage III unresectable NSCLC. Reduced RT followed by sequential chemo-immunotherapy showed similar survival outcomes to the standard RT group without demonstrating equivalence, and reduced severe toxicities, supporting its use in vulnerable populations. However, because the study was not powered for non-inferiority, we cannot conclude that the two regimens are equivalent.

## Supporting information

S1 Approval Clinical trial ethics committee approval form. (PDF)

S1 Protocol The protocol of current trial. (PDF)

S2 Protocol Protocol in original language. (DOCX)

S1 CONSORT Checklist The checklist per the CONSORT (Consolidated Standards of Reporting Trials) 2025 guideline. Licensed under CC BY 4.0. *Hopewell S, Chan AW, Collins GS, Hróbjartsson A, Moher D, Schulz KF, et al. CONSORT 2025 Statement: updated guideline for reporting randomised trials. BMJ. 2025; 388:e081123. https://dx.doi.org/10.1136/bmj-2024-081123*.
(DOCX)

S1 CONSERVE-CONSORT Checklist CONSERVE‑CONSORT checklist detailing modifications due to the COVID‑19 pandemic and their reporting, in accordance with the CONSERVE 2021 statement. (DOCX)

S1 Fig Kaplan–Meier Curves for Progression-Free Survival (PFS) and overall survival (OS) in the per protocol set. Abbreviations: RT, radiotherapy; Note: Survival curves were estimated using the Kaplan–Meier method. Between-group comparisons were descriptive only due to the non-comparative design.
(TIF)

## Abbreviations

AEs: adverse events
CCI: Charlson Comorbidity Index
cCRT: concurrent chemoradiotherapy
CI: confidence interval
COPD: chronic obstructive pulmonary disease
DSMB: Data Safety Monitoring Board
ECOG: Eastern Cooperative Oncology Group
EGFR: epidermal growth factor receptor
ESMO: European Society for Medical Oncology
HR: hazard ratio
ICIs: immune checkpoint inhibitors
ITT: intention-to-treat
JCOG: Japan Clinical Oncology Group
NCCN: National Comprehensive Cancer Network
NSCLC: non-small-cell lung cancer
ORR: objective response rate
OS: overall survival
PFS: progression-free survival
PS: Performance Status
PPS: Per-Protocol Set
RT: radiotherapy
SCRT: sequential chemoradiotherapy
