# Supplementary material for: Sequential chemo-immunotherapy followed by standard versus reduced thoracic radiotherapy for older and/or frail stage III non-small-cell lung cancer: A randomized open-label cohort trial
Source: PLoS Med. 2026 May 27;23(5):e1005111. doi: 10.1371/journal.pmed.1005111 (PMC13215528; doi:10.1371/journal.pmed.1005111)
Supplement: S1 Approval — (PDF) [file pmed.1005111.s001.pdf]

涉及人体科研项目伦理委员会审批件
CLINICAL TRIAL ETHICS COMMITTEE APPROVAL FORM

伦理委员会编号<br>ETHICS COMMITTEE REFERENCE NUMBER:	（2021）临伦审第（189）号
研究方案名称<br>STUDY TITLE:	针对无法耐受同期放化疗的体弱和/或老年III期非小细胞肺癌患者的最优放疗剂量探索-一项开放的、单中心、单臂、双队列的前瞻性临床研究
主要研究者/科室<br>PRINCIPAL INVESTIGATER /DEPARTMENT:	赵胜光 项轶 / 放射治疗科 呼吸与危重症医学科
科研项目资助及编号<br>SPONSORED BY PROJECT / NO.	自选课题（自筹）

下面划[√]的研究相关文件已经审阅
The following items [√] have been reviewed in connection with the above study to be conducted by the above Investigator:

- [√] 研究方案 **Clinical Trial Protocol** 1.0/2021-01-27
- [√] 知情同意书 **Patient Information and Informed Consent Form** 1.0/2021-01-27
- [ ] 受试者招募广告 **Advertisement For Recruitment**
- [ ] 不良事件报告 **Serious Adverse Event**
- [√] 其他 **Other** CRF（1.0/2021-01-27）

审阅结果
And have been:

- [ ] 无条件通过 **Approved**
- [√] 有条件通过（请指出修改意见）**Conditionally Approved (identify item and specify modification below or in accompanying letter)**
- [ ] 拒绝（请指出原因）**Rejected（indentify item and specify reasons below or in accompanying letter）**

评价
Comm[illegible]的研究方案及相关文件经伦理委员会审查，基本符合伦理规范，请对下述内容作相应修改后，同意进行临床研究：请补充退出受试者处理预案。

[signature] 2021.7.9

Signature of Chairman **Date(DD/MMM/YYYY)**

地址：上海市瑞金二路 197 号
邮编：200025
No.197 Ruijin Er Road
Shanghai, P.R. China 200025
