# Supplementary material for: Sequential chemo-immunotherapy followed by standard versus reduced thoracic radiotherapy for older and/or frail stage III non-small-cell lung cancer: A randomized open-label cohort trial
Source: PLoS Med. 2026 May 27;23(5):e1005111. doi: 10.1371/journal.pmed.1005111 (PMC13215528; doi:10.1371/journal.pmed.1005111)
Supplement: S1 Protocol — (PDF) [file pmed.1005111.s002.pdf]

Standard vs Optimized Thoracic Radiotherapy followed by sequential chemo-immunotherapy for Elderly And/or Frail Stage III Non-small-cell Lung Cancer: a randomized open-label cohort trial

Clinical study protocol

Project No: NCT05557552

Sponsor: Ruijin Hospital, Shanghai jiaotong university school of medicine, China

Principal Investigator: Shengguang Zhao and Yi Xiang

Version:2.0

Date: August 11, 2021

Protocol Synopsis

Name	Standard vs Optimized Thoracic Radiotherapy followed by sequential chemo-immunotherapy for Elderly And/or Frail Stage III Non-small-cell Lung Cancer: a randomized open-label cohort trial
Edition/date	Edition 2.0/Aug.11, 2021
Sponsor	Ruijin Hospital, Shanghai jiaotong university school of medicine, China
Principal Investigator	Shengguang Zhao and Yi Xiang
Study type	Investigator-initiated trial
Study	Elderly And/or Frail Stage III Non-small-cell Lung Cancer
Study objective	To investigate the effectiveness and safety of sequential chemoradiotherapy followed by immune checkpoint inhibitor maintenance therapy in locally advanced Elderly And/or Frail non-small-cell lung cancer who cannot tolerate concurrent chemoradiotherapy. Additionally, we aim to assess the clinical efficacy and safety of a reduced dose of thoracic radiotherapy in this population.
Study design	Prospective randomized open-label, cohort study
Estimated enrollment	56 patients
First inclusion criteria	1. Age ≥18 years at time of study entry<br>2. Histologically documented diagnosis of unresectable stage III NSCLC<br><br>Note: For non-squamous cell carcinoma: subjects with known positive EGFR sensitive mutation must be excluded.<br><br>3. Fully-informed written consent obtained from patients

	4. Unfit for concurrent chemoradiotherapy as determined by the multi-disciplinary team board due to one of the following reasons: (1) ECOG PS of 2; (2) Age≥70 with ECOG PS 0-1; (3) Age≥65 with CCI 1<br>5. Adequate bone marrow, liver and kidney function<br>6. Life expectancy of at least 3 months<br>7. At least one measurable (RECIST 1.1), thoracic lesion that can be irradiated<br>8. Histologic or cytologic confirmation of non-small cell lung cancer<br>9. Adequate pulmonary function with FEV1 >1 L or >30 % of predicted value and DLCO >30 % of predicted value
Exclusion criteria	1. Previous chemo-, immuno- or radiotherapy for NSCLC<br>2. Major surgical procedure last 28 days<br>3. History of allogenic organ transplantation, autoimmune disease, immunodeficiency, hepatitis or HIV<br>4. Uncontrolled intercurrent illness<br>5. Other active malignancy<br>6. Leptomeningeal carcinomatosis<br>7. Immunosuppressive medication<br>8. Pregnant or breastfeeding women
Second inclusion criteria	The treatment response is evaluated as complete response, partial response or stable disease after 4-6 cycles of chemo-immunotherapy according to RECIST 1.1;
Study drug dose	**Induction phase：** 4-6 cycles of platinum-based two-drug combination

/route administration	regimen combined with ICIs (immune checkpoint inhibitors);<br>Acceptable chemotherapy regimens include Etoposide/Vinorelbine/Paclitaxel/Docetaxel/Pemetrexed + platinum, with the exclusion of Gemcitabine.<br>**EP Regimen**<br>• Etoposide: 50 mg/m², Days 1–5;<br>• Cisplatin: 50 mg/m², Days 1, 8;<br>**TP Regimen**<br>• Paclitaxel/nab-paclitaxel: 135–175 mg/m², Day 1<br>• Cisplatin: 75 mg/m², Day 1 or Carboplatin: AUC=5 mg/mL/min; D1<br>**DP Regimen**<br>• Docetaxel: 75 mg/m², Day 1<br>• Cisplatin: 75 mg/m², Day 1 or Carboplatin: AUC=5 mg/mL/min; D1<br>**AP Regimen**<br>• Pemetrexed: 500 mg/m², Day 1<br>• Cisplatin: 75 mg/m², Day 1 or Carboplatin: AUC=5 mg/mL/min; D1<br>Acceptable ICIs included ati-PD-1 (pembrolizumab 200mg q.3.w; **Nivolumab 240mg q.3.w.;** Sintilimab 200mg q.3.w.; toripalimab 200mg q.3.w.; Camrelizumab 200mg q.3.w.; tislelizumab 200mg q.3.w.) and anti-PD-L1 (durvalumab, 1000mg q.3.w; Atezolizumab 1200mg q.3.w. ; sugemalimab 1200mg q.3.w.)<br>**Concurrent RT phase**： Frail/elderly stage III NSCLC patients were randomized to receive ICIs combined with either standard thoracic radiotherapy dose 60Gy/30Fx (standard group cohort A) or optimized thoracic radiotherapy dose 50Gy/25Fx (optimized group cohort B).

	**ICIs maintenance phase**： ICIs maintenance at least 6 months or until disease progression or unacceptable toxicity or death;
**Study endpoint**	**Primary endpoint:**<br>(1)1-year progression-free survival: evaluated by investigators according to Response Evaluation Criteria in Solid tumors (RECIST) v.1.1<br>Secondary endpoints:<br>(1) Overall survival;<br>(2) objective response rate evaluated by investigators according to RECIST 1.1 version<br>(3) incidence of treatment-related toxicities;
Sample size and statistical analysis	This study was designed using Simon's two-stage statistical method. Assuming an improvement in the 1-year progression-free survival (PFS) rate from 20% to 40% after maintenance immunotherapy with sequential chemoradiotherapy in patients with stage III non-small cell lung cancer (NSCLC) who were unable to tolerate concurrent chemoradiotherapy, cohort A and cohort B were each projected to enroll 25 patients. After accounting for a 10% patient dropout rate, a total of 56 patients (28 in each cohort) were required to be enrolled.<br>Continuous data were reported as median (IQR or range), and categorical data were reported as frequency (percentage). Survival was estimated using the Kaplan–Meier method, and the 95% confidence interval (CI) of survival was estimated using the Brookmeyer-Crowley method.

Table of Contents

0.PROTOCOL SYNOPSIS 2

0.1 schema... 2

0.2 objectives 2

0.3 subjects 2

0.3.1 eligibility criteria for the first registration 2

0.3.2 eligibility criteria for the second registration 3

0.4 exclusion criteria for the second registration 3

0.5 treatment 4

0.6 study endpoint.. 5

0.7 sample size and statistical analysis.. 5

1 background and rationale for the study plan.................................... 9

1.1 epidemiology for non-small-cell lung cancer 9

1.2 the role of ICIs in NSCLC.. 9

1.3 Treatment challenge for older and/or fragile patients........................ 10

1.4 The rationale for the study... 11

2.0 Research objective and endpoints... 12

2.1 study objective 12

2.2 primary endpoint.. 12

2.3 Secondary endpoint.. 13

3.0 study design.. 13

4. Patient enrollment and exit…………………………………………………… 14

4.1 Inclusion Criteria for first registration ……………………………………… 14

4.2 Major Exclusion Criteria …………… ……………………………………… 15

4.3 Inclusion criteria for second registration………………………………… 15

4.4 Exit criteria…………………………………………………………………… 15

4.5 Termination criteria………………………………………………………… 16

5. Radiation therapy regimen…………………………………………………… 16

6.0 Drug therapy ………………………………………………………………… 18

6.1 Induction chemo-immunotherapy phase…………………………………… 28

6.2 concurrent radiotherapy phase…………………………………………… 19

6.3 ICIs maintenance phase…………………………………………………… 20

7. DOSE ADJUSTMENT for ICIs …………………………………………… 20

7.1.1 ICIs adjustment……………………………………………………… 20

7.1.2 Criterial for ICIs withholding…………………………………………… 20

7.1.3 Criterial for resuming ICIs treatment……………………………… 21

7.1.4 Criteria for permanent discontinuation of ICIs……………………… 22

7.2 DOSE MODIFICATION OF CHEMOTHERAPY…………………… 24

7.2.1 pemetrexed+ carboplatin/cisplatin……………………………………… 25

7.2.2 Paclitaxel/nab-paclitaxel/docetaxel/ Etoposide + carboplatin/cisplatin combination……………………………………………………………………… 27

7.2.2.1 Hematologic toxicity……………………………………………………… 27

7.2.2.2 non-hematological toxicity……………………………………………… 28

8. Laboratory test and tumor response evaluation ………………………… 29

8.1 tests before the first registration.. 29

8.2 tests and evaluation before the second registration.......................... 30

8.2.1 Patient basic information.. 30

8.3 Evaluation During the Induction chemo-immunotherapy phase......... ... 31

8.4 Evaluation during ICIs Maintenance Treatment................................ 31

9 Safety Evaluation.. 32

10. Data analysis and statistical methods 32

10.1 Sample size calculation.. 32

10.2 Analysis Set.. 33

10.3 Treatment of missing values.. 33

10.4 General principles of statistical analysis................................... 33

10.4.1 Patients distribution and dropouts... 34

10.4.2 Demographics and Baseline Characteristics................................ 34

APPENDICES...36

1. Background and rationale for the study plan

1.1 Epidemiology for non-small cell lung cancer

Lung cancer is a significant public health issue, with an estimated 2.2 million new cases and 1.8 million deaths in 2020 worldwide[1]. Non-small-cell lung cancer (NSCLC) is a prevalent form of lung cancer, accounting for approximately 85% of all cases. Stage III NSCLC, also known as locally advanced lung cancer, makes up 15-20% of all lung cancers, and can be categorized into resectable and unresectable subtypes. Unfortunately, the majority of patients with stage IIIA/B (N2) and IIIc disease have lost the chance for curative intent with surgery, making radical concurrent chemoradiotherapy the standard of care for this population[2, 3]. However, patients receiving this regimen have a median disease progression free survival of only 8-10 months and a 5-year survival rate of only 15-20%. Attempts to improve the efficacy of radical concurrent chemoradiotherapy, such as increasing induction and consolidation chemotherapy, and increasing radiotherapy dose, have largely been unsuccessful[4, 5].

1.2 The role of ICIs in NSCLC

In recent years, immune checkpoint inhibitors have emerged as a promising treatment option for unresectable stage III NSCLC[6, 7]. The Pacific study, which administered immune consolidation therapy following radical concurrent chemoradiotherapy, showed significant improvements in both progression-free and overall survival times for stage III NSCLC, breaking the bottleneck in the efficacy of radical concurrent chemoradiotherapy for nearly 20 years[8]. The study demonstrated that patients with locally advanced unresectable stage III NSCLC who did not experience disease progression after receiving standard platinum-containing regimens of concurrent chemoradiotherapy had a median progression-free survival of 16.9 months and a five-year overall survival rate of 42.9% in the durvalumab group compared to 5.6months and 33.4% in the placebo group. Based on these results, the current NCCN guidelines for stage III unresectable NSCLC recommend that patients

who do not progress after radical concurrent chemoradiotherapy continue to be treated with durvalumab immune maintenance.

1.3 Treatment challenge for older and/or fragile patients

Many countries around the world are facing the challenge of an aging society, with a significant increase in the number of elderly oncology patients in recent years. According to statistics from Europe, America, and China, more than 60% of new cancer cases and over 70% of cancer deaths occur in people over 65 years of age[9]. However, there is still controversy surrounding the use of radical concurrent chemoradiotherapy in elderly or frail patients with stage III NSCLC. A study by American scholar Stinchcombe et al reviewed data from 16 clinical trials conducted between 1990 and 2012[10]. The study analyzed 2768 NSCLC patients under the age of 70 as a control group, and 832 NSCLC patients over the age of 70 as a study group, all of whom had inoperable locally advanced NSCLC and received concurrent chemoradiotherapy. The study found that patients over 70 years of age had worse survival rates (OS: 1.20, 95% CI: 1.09-1.31; PFS: 1.17, 95% CI: 1.07-1.29, figure 1) and significantly more grade 3 toxicities (OR 1.35, 95% CI: 1.07-1.70). Furthermore, elderly patients had lower completion rates of concurrent chemoradiotherapy (47% vs. 57%, $P < 0.01$), higher rates of interrupted treatment (20% vs. 13%, $P < 0.01$), and increased on-treatment mortality (7.8% vs. 2.9%, $P < 0.01$). Therefore, radical concurrent chemoradiotherapy regimens are not recommended for elderly or frail patients with locally advanced

NSCLC.

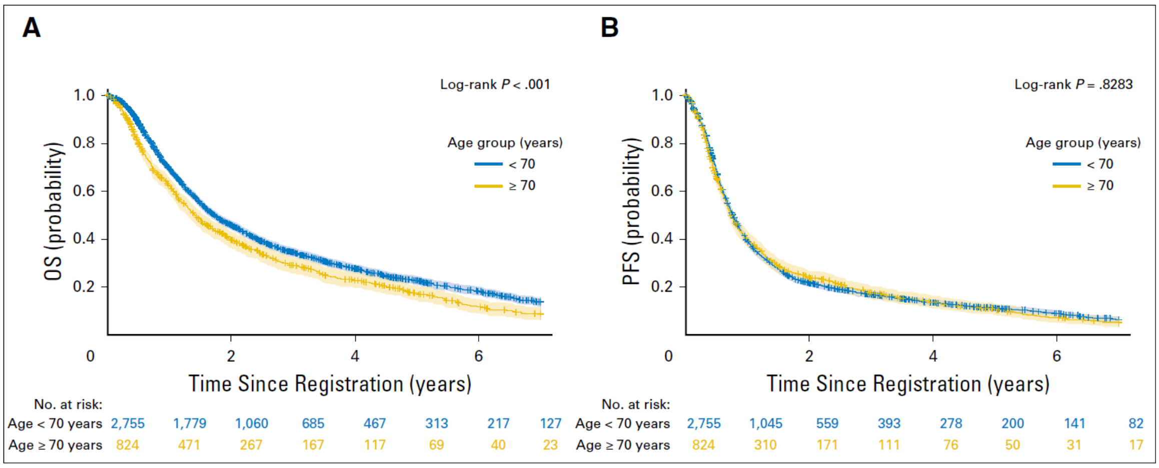

Fig 2. Kaplan-Meier curves for (A) overall survival (OS) and (B) progression-free survival (PFS); product-limit survival estimates based on LIFETEST procedure in SAS software. Crosses indicate censored patients; shaded areas indicate 95% CIs.

Figure 1 survival comparison between <70 years cohort and ≥70 years after definitive CCRT

1.4 The rationale for the study

Additionally, a subgroup analysis of the Pacific study revealed that a certain group of patients received less than 60 Gy of radiotherapy due to their large tumor burden making it difficult to tolerate a higher dose[11]. This subset of patients typically had a poorer prognosis due to their larger tumor burden and lower radiotherapy dose. However, the immunotherapy group showed consistent benefits in terms of progression-free survival (PFS) and overall survival (OS), with an additional advantage seen in the OS of those receiving lower radiotherapy doses (less than 60-66 Gy). In fact, nearly half of patients in clinical settings cannot tolerate 60 Gy doses, and a larger number of patients could benefit from this treatment approach if those with lower radiotherapy doses can benefit from combined immune consolidation therapy. Therefore, our study aimed to establish a cohort of elderly patients receiving low-dose irradiation (50Gy / 25Fx) as part of immune radical sequential radiotherapy group in order to explore the clinical efficacy and safety of immune combination therapy with low-dose sequential chemoradiotherapy (figure 2).

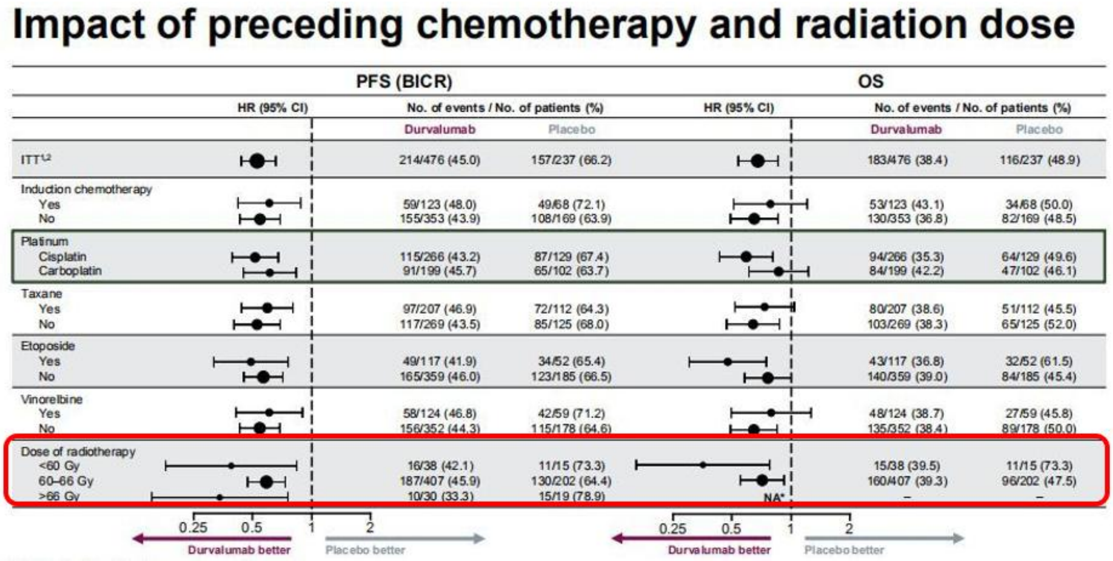

Figure 2 sub-group analysis according to RT dose in PACIFIC Trial

Based on the aforementioned research background, our research group plans to conduct a prospective single-arm clinical study with two cohorts to investigate the effectiveness and safety of sequential chemoradiotherapy combined with immune maintenance therapy in individuals who cannot tolerate concurrent chemoradiotherapy. Additionally, we aim to assess the clinical efficacy and safety of a reduced dose of thoracic radiotherapy in this population.

2.0 Research objective and endpoints

2.1 Study objective

This is a phase II randomized cohort study to explore the efficacy and safety of the chemoimmunotherapy followed by standard or optimized thoracic radiotherapy and ICIs maintenance therapy in untreated patients with unresectable stage III NSCLC.

2.2 primary endpoint

The primary endpoint: 1-year progression-free survival of the two cohorts received sequential chemoradiotherapy plus ICIs maintenance therapy.

Progression-free survival (PFS) will be defined as the duration from the randomization until disease recurrence or progression at any site. For patients who are lost to follow-

up or have not yet passed away at the end of the follow-up period, their data will be censored at the last available follow-up.

2.3 Secondary endpoint

Overall survival, which was defined as the time from randomization to death from any cause. Data for patients lost to follow-up or not yet deceased at the end of follow-up will be censored at the last follow-up for that patient.

Objective response rate (ORR), the best ORR was evaluated by RECIST 1.1

Incidence of treatment-related toxicities, which was defined as the incidence of radiotherapy-related acute toxicity of grade III and above (according to CTCAE4.03) and subacute toxicity (RTOG) within 3 months after completion of radiotherapy using different doses in both groups. The safety of this treatment regimen, including long-term toxicity (RTOG), was also assessed.

3.0 study design

Our trial is an open, single-center, randomized, two-cohort prospective clinical study aimed at determining the efficacy and safety of optimal radiotherapy dose for frail and/or elderly patients with unresectable stage III NSCLC who can not tolerate concurrent chemoradiotherapy (Figure 3).

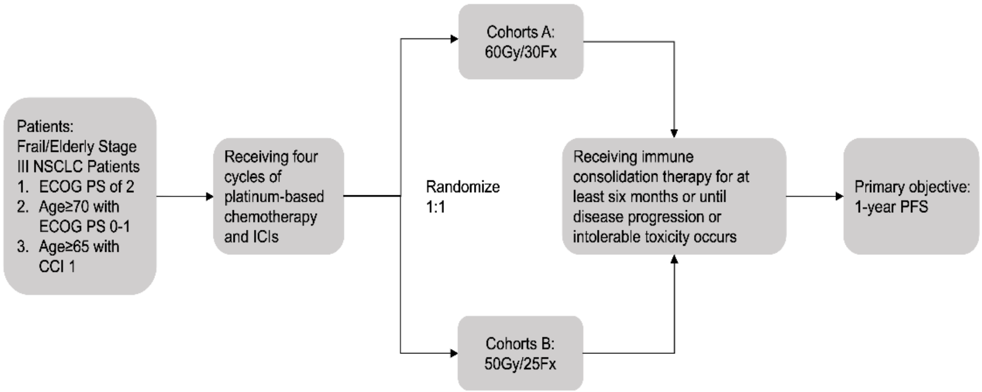

Figure 1: Study design. CCI: Charlson Comorbidity Index; ECOG PS: Eastern cooperative oncology group performance status; ICIs: immune checkpoint inhibitors; NSCLC: non-small cell lung cancer; PFS: progression-free survival.

(1) Inductive chemo-immunotherapy phase: Both cohort A and cohort B will receive four-six cycles of platinum-based two-drug combination chemotherapy regimens combined with ICIs.

(2) Concurrent radiotherapy-immunotherapy phase: Radiotherapy regimen: Patients who have shown no disease progression after chemo-immunotherapy will receive photon radiation within six weeks of the last administration of the systematic therapy. Patients would be randomized to 60 Gy/30 Fx for cohort A and 50 Gy/25 Fx for cohort B;

(3) ICIs maintenance phase: ICIs maintenance at least 6 months (8cycles) or until disease progression or unacceptable toxicity or death;

4. Patient enrollment and exit

4.1 Inclusion Criteria for first registration

1)Age ≥18 years at time of study entry

2)Histologically documented diagnosis of unresectable stage III NSCLC;

Note: For non-squamous cell carcinoma: subjects with known positive EGFR sensitive mutation must be excluded.

3)Fully-informed written consent obtained from patients

4)Unfit for concurrent chemoradiotherapy as determined by the multi-disciplinary team board due to one of the following reasons: (1) ECOG PS of 2; (2) Age≥70 with ECOG PS 0-1; (3) Age≥65 with CCI 1

5)Adequate bone marrow, liver and kidney function

6)Life expectancy of at least 3 months

7)At least one measurable (RECIST 1.1), thoracic lesion that can be irradiated

8) Histologic or cytologic confirmation of non-small cell lung cancer

9) Adequate pulmonary function with FEV1 >1 L or >30 % of predicted value and DLCO >30 % of predicted value

4.2 Major Exclusion Criteria

1)Previous chemo-, immuno- or radiotherapy for NSCLC

2)Major surgical procedure last 28 days

3)History of allogenic organ transplantation, autoimmune disease, immunodeficiency, hepatitis or HIV

4)Uncontrolled intercurrent illness

5)Other active malignancy

6)Leptomeningeal carcinomatosis

7) Immunosuppressive medication

8) Pregnant or breastfeeding women

4.3 Inclusion criteria for second registration

1) The treatment response is evaluated as complete response, partial response or stable disease after 4-6 cycles of chemo-immunotherapy according to RECIST 1.1;

2) agree to received thoracic radiotherapy and ICIs maintenance therapy;

4.4 Exit criteria

Subjects can withdraw their informed consent and exit the trial at any time. The investigator may decide to withdraw the subject from the study under the following circumstances:

1) Any clinical adverse event, laboratory abnormality, pregnancy event, or other medical condition that may no longer benefit the subject.

2) Subjects meet any exclusion criteria and may not be able to participate further in the trials (including newly developed clinical indications during the trial or persistent antiquated problems that could not be detected in time

3) From the perspective of medical ethics, it is deemed necessary to stop the test.

4) Subjects with poor compliance, no longer receiving medication or testing before completing all trials, or receiving other anti-tumor treatments at the same time before the completion of the trial, and unable to adhere to the completion of the trial as planned.

4.5 Termination criteria

This study may be terminated or suspended early if there are sturdy reasons. Written notice setting out the reasons for the early termination or suspension will be provided by the deciding party and sent to the investigator, sponsor, ethics committee, and relevant departments. The reasons for termination of this study include but are not limited to the following:

1) Major errors in the clinical trial protocol were found in the trial, making it difficult to evaluate the treatment results;

2) The sponsor requests termination;

3) The relevant department or the ethics committee ordered the termination of the trial for some reason.

5. Radiation therapy regimen

All patients should be evaluated in the Multi-Disciplinary Treatment team to determine their eligibility for radiotherapy and to be informed of the potential toxicity and prognosis of the treatment before proceeding with body position fixation, positioning CT, planning, and treatment. Patients meeting the inclusion criteria will receive intensity-modulated radiation therapy / Volumetric Modulated Arc Therapy targeting the involved field of the tumor lesion, including the primary lesion and metastatic lymph nodes, with a dose fractionation of 60Gy / 30Fx for cohort A and 50Gy / 25Fx for cohort B.

Individualized postural fixation devices were designed according to the radiation field requirements, with patients positioned in the supine position with both arms raised over the head. A wing board, vacuum pads, and footpads were used to secure the patient's position. A chest enhanced positioning CT was performed with the scan scope suggested from the neck to the lower margin of the kidneys including the tumor and adjacent vital organs as per the radiation field requirements.

The clinician delineated the gross tumor volume (GTV) on each slice of the localizing CT, including the primary lesion and metastatic lymph nodes. Furthermore, to account for respiratory motion, a 4D CT scan was used for lung cancer patients to generate an internal gross tumor volume (iGTV) that encompasses the tumor motion throughout the breathing cycle. The clinical tumor volume (CTV) was formed by externally expanding the GTV by 6 mm for squamous carcinoma and 8 mm for adenocarcinoma, including the lymph drainage areas with metastatic lymph nodes while not extending beyond anatomical boundaries. The planning target volume (PTV) was defined as the CTV plus 0.5 cm in all directions.

The radiation treatment was delivered in a 1-day, 5-weekly schedule. Dose optimization aimed to achieve 95% prescribed dose coverage of the PTV volume while keeping the dose to normal organs within tolerable limits. The PTV maximum dose was not allowed to exceed 107% of the prescribed dose, with 105% - 107% of the prescribed dose covering no more than 5% of the PTV volume.

The normal tissue volumes contoured for organs at risk (OAR) were the lung, esophagus, spinal cord and heart. The dual lung Dmean≤ 18 Gy, lung V20 ≤30%, and lung V5≤65%, while the esophageal Dmean was kept below 30 Gy. The cardiac V40 was kept below 30%, and the cardiac Dmean was kept below 24 Gy.

Patients undergoing treatment might require plan changes due to body shape changes, tumor regression, or side effects. Any plan changes should be discussed with the attending physician, with reasons and further treatment measures promptly recorded. Patients should continue to be followed up for toxicities and efficacy.

6.0 Drug therapy

6.1 Induction chemo-immunotherapy phase

Immune checkpoint inhibitors:

Administration: Intravenous infusion (IV) of ICIs over a period of 60 minutes or greater on Day 1 of each treatment cycle, every 3 weeks (21 days, Q3W).

Acceptable ICIs included ati-PD-1 (pembrolizumab 200mg q.3.w; **Nivolumab 240mg q.3.w.;** Sintilimab 200mg q.3.w.; toripalimab 200mg q.3.w.; Camrelizumab 200mg q.3.w.; tislelizumab 200mg q.3.w.) and anti-PD-L1 (durvalumab, 1000mg q.3.w; Atezolizumab 1200mg q.3.w.; sugemalimab 1200mg q.3.w.)

Chemotherapy

ICIs will be administered firstly, and chemotherapy may be started 30 minutes after the completion of ICIs, if they are given on the same day. Acceptable chemotherapy includes Etoposide/Vinorelbine/Paclitaxel/Docetaxel/Pemetrexed + platinum for 4-6 cycles, with the exclusion of Gemcitabine.

EP Regimen

- Etoposide: 50 mg/m², Days 1–5;
- Cisplatin: 50 mg/m², Days 1, 8;

TP Regimen

- Paclitaxel/nab-paclitaxel: 135 mg/m², Day 1
- Cisplatin: 75 mg/m², Day 1 or Carboplatin: AUC=5 mg/mL/min; D1

DP Regimen

- Docetaxel: 75 mg/m², Day 1
- Cisplatin: 75 mg/m², Day 1 or Carboplatin: AUC=5 mg/mL/min; D1

AP Regimen

- Pemetrexed: 500 mg/m², Day 1
- Cisplatin: 75 mg/m², Day 1 or Carboplatin: AUC=5 mg/mL/min; D1

Pretreatment for pemetrexed + platinum chemotherapy (table 1)

**Pretreatment**	**Dose/route of administration**	**Duration**
Folic acid (or other Compound vitamin Products containing folic acid)	350-100 μg PO	Administered once daily from at least 5-7 days before Cycle1 Day<br>1until 3 weeks after discontinuation of pemetrexed
Vitamin B12	1000 μg IM	Administered intramuscularly once within 7 days prior to Cycle1<br>Day1, and then administered intramuscularly once every 3 cycles.<br>Subsequent vitamin B12 could be given on the same day of<br>pemetrexed administration.
Dexamethasone (or drugs of the same class)	4 mg PO	Twice a day, one day before, on the day of and one day after each<br>pemetrexed infusion.

IM=intramuscular; PO=oral.

Note: Prophylactic antemetic is given according to local guidance.

6.2 concurrent radiotherapy phase

Frail/elderly stage III NSCLC patients were randomized to receive ICIs combined with either standard thoracic radiotherapy dose 60Gy/30Fx (standard group cohort A) or optimized thoracic radiotherapy dose 50Gy/25Fx (optimized group cohort B). Concurrent ICIs will be administered during radiotherapy every 21 days;

6.3 ICIs maintenance phase

After thoracic radiotherapy, ICIs maintenance would be continued at least 6 months (8 cycles) or until disease progression or unacceptable toxicity or death;

7. DOSE ADJUSTMENT for ICIs

7.1.1 ICIs adjustment

Dose adjustment is not permitted for ICIs. In case of any treatment-realted adverse event, the ICIs should be withheld or discontinued according to the criteria below. The withholding of ICIs should not exceed 12 weeks.

7.1.2 Criterial for ICIs withholding

Any grade 2 drug-related adverse event (non-dermalogical event), except for the following situations:

– For grade 2 drug-related fatigue or abnormal laboratory test result, study treatment doesn't need to be postponed.

• Any grade 3 drug-related dermalogical adverse event

• Any grade 3 drug-related abnormal laboratory test result except for the following situations:

– For grade 3 lymphocytopenia, study treatment doesn't need to be postponed.

– Study treatment will be postponed when ≥ grade 2 drug-related toxicity occurs in

a subject whose baseline AST, ALT or total bilirubin level is within normal range.

– Study treatment will be postponed when ≥ grade 3 drug-related toxicity occurs in a subject whose baseline AST, ALT or total bilirubin level is ≤ grade 1 toxicity.

7.1.3 Criterial for resuming ICIs treatment

When a drug-related AE is estimated to resolve to ≤ grade 1 or baseline level within 9 weeks of the planned dose (12 weeks of the last dose), ICIs may resume except for the following situations:

- A subject may resume treatment if there's grade 2 fatigue.
- A subject without any grade 3 drug-related dermalogical AE may resume treatment if grade 2 dermalogical toxicity remains.
- A subject may resume treatment despite grade 2 AST/ALT or total bilirubin abnormality occurs, if subject's baseline data reveals grade 1 AST/ALT or total bilirubin abnormality. But if the treatment withholding is due to other causes, the treatment resuming in a subject with grade 2 AST/ALT or total bilirubin abnormality will be reevaluated.
- Subjects with AST/ALT and total bilirubin abnormality that meets the criterion of permanent discontinuation **(refer to Section 7.1.4)** should discontinue study treatment permanently.
- Study treatment cannot be resumed until fully recovery of drug-related pulmonary toxicity, diarrhea or colitis to baseline level. If grade 1 pneumonitis persists in a subject after corticosteroid tapering over more than 1 month, the investigator may resume his/her treatment after consultation with and approval from the sponsor's medical monitor.

• For drug-related endocrinologic disease that can be well controlled by physiologic hormone replacement therapy, the investigator may resume the subject's treatment after consultation with and approval from the sponsor's medical monitor.

Three dose interruption due to toxicity are allowed. In the event of a fourth toxicity requiring dose interruption, the study treatment will be permanently discoutinued after consultation with the sponsor. Please refer to Section 7.1.4 for details.

7.1.4 Criteria for permanent discontinuation of ICIs

ICIs will be permanently discontinued if any of the following drug-related adverse events occurs.

• Grade 2 drug-related uveitis, ophthalmodynia or blurred vision that cannot recover to grade 1 within the recovery time window despite topical treatment or needs systemic treatment.

• Grade 3 or more severe infusion-related reaction during or after ICIs infusion.

• Any grade 3 non-dermalogical drug-related adverse event that lasts for > 7 days, except for the following situations.

– A subject who develops grade 3 drug-related uveitis, pneumonitis, bronchospasm, and hypersensitivity or infusion-related reaction must discontinue treatment regardless the duration of adverse event.

– A subject with grade 3 drug-related endocrinologic disease that can be well controlled by physiologic hormone replacement therapy doesn't need to discontinue study treatment.

– A subject with grade 3 drug-related abnormal laboratory test result doesn't need to discontinue treatment except for the following situations:

(1) A subject with grade 3 drug-related thrombocytopenia that lasts for > 7 days or has associated bleeding event should discontinue study treatment.

(2) A subject with any of the following abnormal liver function test (LFT) should discontinue study treatment:

– AST or ALT > 5-10 x ULN for > 2 weeks

– AST or ALT > 10 x ULN

– Total bilirubin > 5 X ULN

– AST or ALT > 3 X ULN and concurrent total bilirubin > 2 X ULN

• Any grade 4 drug-related adverse event or abnormal laboratory test result except for the following situations:

– Grade 4 neutropenia lasting for ≤ 7 days

– Grade 4 lymphocytopenia or leukopenia

– For solitary grade 4 amylase or lipase abnormality without any symptom or sign of pancreatitis, when a grade 4 amylase or lipase abnormality occurs, the investigator should consult sponsor's medical monitor.

– Solitary grade 4 electrolyte disorder/abnormality that can be corrected by electrolyte supplementation/adequate treatment in 72 hours without any clinical consequence.

– A subject with grade 4 drug-related endocrinologic adverse events that can be resolved or well controlled by physiologic hormone (for example corticosteroid, thyroid hormone) replacement treatment, for example, adrenal insufficiency, ACTH

deficiency, hyperthyroidism or hypothyroidism or decreased sugar tolerance, doesn't need to discontinue treatment with communication with and approval from sponsor's medical monitor first.

• Any event that causes the next dosing > 12 weeks from the last dose requires study drug discontinuation, except for any of the following situations:

7.2 DOSE MODIFICATION OF CHEMOTHERAPY

The dose modification of chemotherapy follows local clinical practice and the package inserts of medications. The investigator may reduce the dose according to the severity of AE occurred after chemotherapy monotherapy or combination therapy, when necessary. The subject is permitted to proceed into the next treatment cycle if the drug-related AE has recovered to ≤ grade 1 or baseline level [or ≤ grade 2 for alopecia, fatigue or other drug-related AEs that per the investigator's judgment will not affect medication safety. Treatment cycle delay due to unresolved AE is allowed. If an AE is caused by a specific drug per the investigator's judgment, and it has caused dose adjustment or treatment delay of this specific drug, the other drug could be administered provided it is not contraindicated.

The reduced dose due to drug-related AE will not be increased. Dose reduction is only permitted twice for each chemotherapy drug. At the third dose reduction, the subject has to withhold the treatment.

If the AE is caused by one chemotherapy drug judged by the investigator, the dose will be modified for this chemotherapy drug only. If the AE are caused by the combination of two chemotherapy drugs, the dose will be modified for both drugs.

The chemotherapy can be withheld for 6 weeks at most. Adverse events and laboratory findings are graded based on NCI CTCAE v4.03. All dose modifications follow the principle of maximum dose modification. Refer to Table 2 for dose modification

instructions

Table 2 Dose modification for drugs

	Dose level 0 (DL-0)	Dose level 1 (DL-1)	Dose level 2 (DL-2)	Dose level 3(DL-3)
Carboplatin	AUC 5<br>Maximum dose: 750 mg	AUC 3.5<br>Maximum dose: 562.5 mg	AUC 2.5<br>Maximum dose: 375 mg	Discontinue
Cisplatin	75 mg/m^2	56 mg/m^2	37.5mg/m^2	Discontinue
Pemetrexed	500 mg/m^2	375 mg/m^2	250 mg/m^2	Discontinue
Paclitaxel/nab-paclitaxel	175 mg/m^2	132 mg/m^2	88 mg/m^2	Discontinue
Docetaxel	75 mg/m^2	56 mg/m^2	37.5mg/m^2	Discontinue
Etoposide	50mg/m^2	37.5mg/m^2	25mg/m^2	Discontinue
ICIs	ati-PD-1 (pembrolizumab 200mg q.3.w; Nivolumab 240mg q.3.w.; Sintilimab 200mg q.3.w.; toripalimab 200mg q.3.w.; Camrelizumab 200mg q.3.w.; tislelizumab 200mg q.3.w.) and anti-PD-L1 (durvalumab, 1000mg q.3.w; Atezolizumab 1200mg q.3.w.; sugemalimab 1200mg q.3.w.)	Dose reductions are not permitted	Dose reductions are not permitted	Dose reductions are not permitted

7.2.1 pemetrexed+ carboplatin/cisplatin

Dose modification of pementrexed due to hematological toxicity is based on the neutrophil count and platelet count measured on Day 1 of the treatment cycle. The dose of the next cycle will be adjusted based on the lowest blood cell count or the

highest severity of non-hematological toxicity before the start of the next dose. The treatment will be delayed until the patient recovers. Restart of treatment should follow the dose modification principles in Table 3 and 4. The actual dose modification will be decided by the investigator according to subject's status.

Table 3 Recommended dose modifications of carboplatin/cisplatin and pemetrexed for subjects with hematological toxicities

		**Carboplatin/cisplatin**	**Pemetrexed**
Platelets	ANC	Table 2 recommended dose level (DL)	
⩾50,000/mm3	⩾500/mm3	DL-0	DL-0
⩾50,000/mm3	<500/mm3	DL-1	DL-1
<50,000/mm3 without bleeding and	Any	DL-1	DL-1
<50,000/mm3 with ⩾ grade 2 bleeding and	Any	DL-2	DL-2
Any value and	<1000/mm3 and fever ⩾38.5℃	DL-1	DL-1

Table 4 Recommended dose modifications of carboplatin/cisplatin and pemetrexed for subjects with non-hematological toxicities

		**Carboplatin/cisplatin**	**Pemetrexed**
Toxicity	CTC grade	Table 2 recommended dose level (DL)	
Nausea or vomiting	Grade 3/4	DL-0	DL-0
Diarrhea	Grade 3/4	DL-0	DL-1
Mucositis	Grade 3/4	DL-0	DL-2
Neurotoxicity	Grade 3/4	DL-1	DL-1
Transaminase elevation	Grade 3/4	DL-2	DL-2
Other non-hematologic toxicity	Grade 3/4	DL-1	DL-1

7.2.2 Paclitaxel/nab-paclitaxel/docetaxel/ Etoposide + carboplatin/cisplatin combination

7.2.2.1 Hematologic toxicity

Dose modification of paclitaxel/nab-paclitaxel/docetaxel/Etoposide due to hematological toxicity is based on the neutrophil count and platelet count measured on Day 1 of the treatment cycle. Before the start of a cycle, ANC and platelet must be ⩾ 1500/mm3 and ⩾100,000/mm3, respectively. The dose of the next cycle will be modified according to the lowest ANC and platelet count in the last cycle. Study treatment will be changed or withheld according to Table 5 for bone marrow suppression. Actions will be taken according to the local standard of care and guideline. Colony stimulation factor is recommended instead of dose reduction to treatment febrile neutropenia or grade 4 neutropenia. Complete blood cell count is measured once a week after chemotherapy withholding because of hematological toxicity, until the count recovers to the lowest permitted value for treatment, after which, the treatment protocol will be followed as usual. The investigator may modify the dose of carboplatin according to dose modification of paclitaxel/nab-paclitaxel.

Table 5 guidance on dose modification of paclitaxel/nab-paclitaxel/Etoposide due to hematologic toxicity

Platelets	ANC	Paclitaxel/nab-paclitaxel/docetaxel/ Etoposide
⩾100,000/mm3 and	⩾1500/mm3	DL-0
<50,000/mm3 or	<500/mm3 or febrile neutropenia	DL-1
<50,000/mm3 the second time or	<500/mm3 or febrile neutropenia the second time	DL-2
<50,000/mm3 the third time	<500/mm3 or febrile neutropenia the	DL-3

or	third time	

7.2.2.2 non-hematological toxicity

Generally, paclitaxel/nab-paclitaxel/docetaxel/Etoposide can be withheld or dose reduced as per investigator's judgment for severe (grade 3 or 4) non-hematological toxicity and nausea/vomiting. For non-hematological toxicity, refer to Table 6 for guidance on dose modification based on severity and grade of the toxicity.

The investigator may modify the dose of carboplatin/cisplatin according to dose modification of paclitaxel/nab-paclitaxel/docetaxel. Paclitaxel/nab-paclitaxel/docetaxel/ Etoposide will be discontinued permanently for ⩾ grade 3 hypersensitivity and ⩾ grade 3 neurotoxicity.

Table 6 **Guidance on non-hematologic dose modifications**

	**Grade 2**	**≥ Grade 3**
At first occurrence	The treatment can be withheld until the toxicity recovers to grade 0−1, after which, DL-0 should be continued if possible	The treatment can be withheld until the toxicity recovers to grade 0−1, after which, DL-1 should be continued if possible
The same toxicity at the second occurrence	The treatment can be withheld until the toxicity recovers to grade 0−1, after which, DL-1 should be continued	The treatment can be withheld until the toxicity recovers to grade 0−1, after which, DL-2 should be continued
The same toxicity at the third occurrence	The treatment can be withheld until the toxicity	Permanent treatment discontinuation DL-3

	recovers to grade 0−1, after which, DL-2 should be continued	
The same toxicity at the fourth occurrence	Permanent treatment discontinuation DL-3	

8. Laboratory test and tumor response evaluation

8.1 tests before the first registration

8.1.1. A detailed medical history and physical examination should be conducted.

8.1.2. Pathological diagnosis of the primary disease is necessary.

8.1.3. The following laboratory tests should be conducted:

(1) Peripheral blood, urine, and fecal routine tests, as well as fecal occult blood tests.

(2) Blood biochemical tests, including liver function tests (ALT, AST, ALP, jaundice index, direct and indirect bilirubin, albumin, globulin, prealbumin, prothrombin time, and r-GT), renal function tests (creatinine, uric acid, etc.), and myocardial zymography.

(3) Tumor markers such as CEA, NSE, SCC, CA125, and cytokeratin 19 fragment.

(4) Tests for syphilis antibodies, HIV, and a full set of hepaciviruses.

(5) Thyroid function tests (TSH, FT3, FT4).

(6) Autoimmune antibody tests, including anti-ro-52 antibody, anti PM-Scl antibody, anti-centromere protein B antibody, anti vas nuclear antigen antibody, anti-double stranded DNA antibody, anti-nucleosome antibody, anti-histone antibody, anti-ribosomal P protein antibody, anti-mito-m2 antibody, and anti RNP / SM antibody.

(7) Cellular immune function status should be evaluated through CD4CD8 positive cells and CD4CD28 positive cells.

(8) Cytokine changes, including IL-6, IL-8, IL-10, and INF-γ, should be assessed.

8.1.4 Imaging examinations should be performed, including contrast-enhanced CT of the chest (or plain CT if there are contraindications to contrast-enhanced CT), cranial MRI, abdominal ultrasound, and bone scan to exclude distant metastasis.

8.1.5 Cardiac function tests, including ECG, cardiac ultrasound, 24-hour Holter examination (for patients with previous heart disease), and coronary angiography (for patients with previous coronary stenosis or cardiac stent placement), should be conducted.

8.1.6 Pulmonary function testing is required.

8.2 tests and evaluation before the second registration

Tests and evaluations will take place before the second registration. Of note, if a subject has the results of tests carried out within the specified period, the test results prior to informed consent are allowed to be used, if the subject agrees to it. The same tests do not have to be carried out after the informed consent is obtained.

8.2.1 Patient basic information.

8.2.2. The following laboratory tests should be conducted:

(1) Peripheral blood, urine, and fecal routine tests, as well as fecal occult blood tests.

(2) Blood biochemical tests, including liver function tests (ALT, AST, ALP, jaundice index, direct and indirect bilirubin, albumin, globulin, prealbumin, prothrombin time, and r-GT), renal function tests (creatinine, uric acid, etc.), and myocardial zymography.

(3) Tumor markers such as CEA, NSE, SCC, CA125, and cytokeratin 19 fragment.

(4) Tests for syphilis antibodies, HIV, and a full set of hepaciviruses.

(5) Thyroid function tests (TSH, FT3, FT4).

(6) Autoimmune antibody tests, including anti-ro-52 antibody, anti PM-Scl antibody, anti-centromere protein B antibody, anti vas nuclear antigen antibody, anti-double stranded DNA antibody, anti-nucleosome antibody, anti-histone antibody, anti-

ribosomal P protein antibody, anti-mito-m2 antibody, and anti RNP / SM antibody.

(7) Cellular immune function status should be evaluated through CD4CD8 positive cells and CD4CD28 positive cells.

(8) Cytokine changes, including IL-6, IL-8, IL-10, and INF-γ, should be assessed.

8.2.3 Imaging examinations should be performed, including contrast-enhanced CT of the chest (or plain CT if there are contraindications to contrast-enhanced CT), cranial MRI, abdominal ultrasound, and bone scan to exclude distant metastasis.

8.3 Evaluation During the Induction chemo-immunotherapy phase

During radiotherapy, patients should be assessed for tolerance of radiotherapy and acute toxicities at least weekly, and the relevant information should be documented. Blood routine should be reviewed every week to observe hematological toxicity, including white blood cells, neutrophil numbers, platelets, and HB. Liver function should be reviewed every two weeks, and patients should be observed for liver toxicity. Patients should be monitored for fever, cough, expectoration, chest tightness, chest pain, and other symptoms, and lung auscultation and imaging studies may be used, if necessary, to exclude radiation pneumonitis. Swallowing should be observed, and patients should be evaluated for esophageal toxicity.

8.4 Evaluation during ICIs Maintenance Treatment

Tests will take place before the administration of ICIs every 21 days. And evaluation would be performed every 9 weeks;

8.4.1. The following laboratory tests should be conducted:

(1) Peripheral blood, urine, and fecal routine tests, as well as fecal occult blood tests.

(2) Blood biochemical tests, including liver function tests (ALT, AST, ALP, jaundice index, direct and indirect bilirubin, albumin, globulin, prealbumin, prothrombin time, and r-GT), renal function tests (creatinine, uric acid, etc.), and myocardial zymography.

(3) Tumor markers such as CEA, NSE, SCC, CA125, and cytokeratin 19 fragment.

(4) Thyroid function tests (TSH, FT3, FT4).

(5) Autoimmune antibody tests, including anti-ro-52 antibody, anti PM-Scl antibody, anti-centromere protein B antibody, anti vas nuclear antigen antibody, anti-double stranded DNA antibody, anti-nucleosome antibody, anti-histone antibody, anti-ribosomal P protein antibody, anti-mito-m2 antibody, and anti RNP / SM antibody.

8.4.2 Imaging examinations should be performed, including contrast-enhanced CT of the chest (or plain CT if there are contraindications to contrast-enhanced CT), cranial MRI, abdominal ultrasound, and bone scan to exclude distant metastasis.

9 Safety Evaluation

During the trial period, the safety of the treatment methods will be evaluated based on adverse event records, laboratory examinations, vital signs, physical examination, KPS score, cardiac ultrasound, and electrocardiogram records. During the trial, the symptoms and signs of the subjects after receiving the drug should be closely observed. Adverse events/reactions should be addressed in a timely and effective manner to ensure the safety and interests of the subjects.

10. Data analysis and statistical methods

10.1 Sample size calculation

This study was designed using Simon's two-stage statistical method. Assuming an improvement in the 1-year progression-free survival (PFS) rate from 20%[12] to 40% after maintenance immunotherapy with sequential chemoradiotherapy in patients with stage III non-small cell lung cancer (NSCLC) who were unable to tolerate concurrent chemoradiotherapy, cohort A and cohort B were each projected to enroll 25 patients. This study employed a 2-stage design with 12 patients enrolled in each cohort in stage 1, and if a 1-year PFS of 16.7% was achieved, patients in stage 2 were enrolled; otherwise, the study enrollment would be discontinued. After accounting for a 10%

patient dropout rate, a total of 56 patients (28 in each cohort) were required to be enrolled.

10.2 Analysis Set

The analysis population includes the full analysis set (FAS), the per-protocol set (PPS), and the safety set (SS).

Full Analysis Set, FAS: Following the intent-to-treat (ITT) principle, all cases that have been enrolled and received treatment are analyzed for efficacy.

Per-Protocol Set, PPS: All patients who meet the trial protocol, have good compliance, have taken at least one cycle of study drugs (excluding patients who have experienced disease progression with substantial evidence after enrollment), have not received prohibited drugs during the trial period, and completed the CRF equirements. No filling is done for missing data. Efficacy results were statistically analyzed using both FAS and PPS.

Safety Analysis Set, SAS: All the enrolled cases receive treatment and all patients with safety records after radiotherapy. This data set is used for safety analysis.

10.3 Treatment of missing values

Efficacy indicators: All main indicators missing due to early withdrawal of patients are recorded as "unassessable" in the analysis. When the calculation includes time variables (such as PFS), the censoring time is obtained by checking the subjects who have undergone imaging evaluation after

discontinuity. No missing values are estimated for baseline and safety data.

For extreme values of laboratory data caused by improper sample processing, the corresponding unplanned visit data will be used in the analysis, or it will be considered missing data and not included in the analysis.

10.4 General principles of statistical analysis

Unless otherwise specified, the data in this study will be summarized as descriptive statistics in accordance with the following general principles.

Measurement data are summarized by means, standard deviation, median, maximum, and minimum; count data are summarized by frequency and percentage; time-to-event data are summarized by Kaplan-Meier method to estimate survival rate and draw survival curve; blood drug concentration data are summarized using mean, standard

deviation, coefficient of variation, median, maximum, and minimum.

10.4.1 Patients distribution and dropouts

The number of cases (percentage) is used to describe the enrollment and completion of subjects. The distribution of cases in each data set. List the medications of dropouts and excluded patients and the reasons for early withdrawal. The distribution of cases in each analysis set.

10.4.2 Demographics and Baseline Characteristics

Compile statistics descriptively for demographic data and baseline characteristics. Measurement data needs to calculate the number of cases, mean, standard deviation, median, minimum and maximum; Calculate the frequency and composition ratio of count data and level.

References:

1. Zheng R, Zhang S, Zeng H, Wang S, Sun K, Chen R, Li L, Wei W, He J: **Cancer incidence and mortality in China, 2016**. *Journal of the National Cancer Center* 2022, **2**(1):1-9.
2. Curran WJ, Jr., Paulus R, Langer CJ, Komaki R, Lee JS, Hauser S, Movsas B, Wasserman T, Rosenthal SA, Gore E *et al*: **Sequential vs. concurrent chemoradiation for stage III non-small cell lung cancer: randomized phase III trial RTOG 9410**. *Journal of the National Cancer Institute* 2011, **103**(19):1452-1460.
3. Evison M, AstraZeneca UKL: **The current treatment landscape in the UK for stage III NSCLC**. *British journal of cancer* 2020, **123**(Suppl 1):3-9.
4. Bi N, Liang J, Zhou Z, Chen D, Fu Z, Yang X, Feng Q, Hui Z, Xiao Z, Lv J *et al*: **Effect of Concurrent Chemoradiation With Celecoxib vs Concurrent Chemoradiation Alone on Survival Among Patients With Non-Small Cell Lung Cancer With and Without Cyclooxygenase 2 Genetic Variants: A Phase 2 Randomized Clinical Trial**. *JAMA network open* 2019, **2**(12):e1918070.
5. Bradley JD, Hu C, Komaki RR, Masters GA, Blumenschein GR, Schild SE, Bogart JA, Forster KM, Magliocco AM, Kavadi VS *et al*: **Long-Term Results of NRG Oncology RTOG 0617: Standard- Versus High-Dose Chemoradiotherapy With or Without Cetuximab for Unresectable Stage III Non-Small-Cell Lung Cancer**. *J Clin Oncol* 2020, **38**(7):706-714.
6. Barlesi F, Vansteenkiste J, Spigel D, Ishii H, Garassino M, de Marinis F, Ozguroglu M, Szczesna A, Polychronis A, Uslu R *et al*: **Avelumab versus docetaxel in patients with platinum-treated advanced non-small-cell lung cancer (JAVELIN Lung 200): an open-label, randomised, phase 3 study**. *Lancet*

Oncol 2018, **19**(11):1468-1479.

7. Hui R, Garon EB, Goldman JW, Leighl NB, Hellmann MD, Patnaik A, Gandhi L, Eder JP, Ahn MJ, Horn L *et al*: **Pembrolizumab as first-line therapy for patients with PD-L1-positive advanced non-small cell lung cancer: a phase 1 trial**. *Annals of oncology : official journal of the European Society for Medical Oncology / ESMO* 2017, **28**(4):874-881.
8. Spigel DR, Faivre-Finn C, Gray JE, Vicente D, Planchard D, Paz-Ares L, Vansteenkiste JF, Garassino MC, Hui R, Quantin X *et al*: **Five-Year Survival Outcomes From the PACIFIC Trial: Durvalumab After Chemoradiotherapy in Stage III Non-Small-Cell Lung Cancer**. *Journal of clinical oncology : official journal of the American Society of Clinical Oncology* 2022, **40**(12):1301-1311.
9. Bray F, Ferlay J, Soerjomataram I, Siegel RL, Torre LA, Jemal A: **Global cancer statistics 2018: GLOBOCAN estimates of incidence and mortality worldwide for 36 cancers in 185 countries**. *CA: a cancer journal for clinicians* 2018, **68**(6):394-424.
10. Stinchcombe TE, Zhang Y, Vokes EE, Schiller JH, Bradley JD, Kelly K, Curran WJ, Jr., Schild SE, Movsas B, Clamon G *et al*: **Pooled Analysis of Individual Patient Data on Concurrent Chemoradiotherapy for Stage III Non-Small-Cell Lung Cancer in Elderly Patients Compared With Younger Patients Who Participated in US National Cancer Institute Cooperative Group Studies**. *J Clin Oncol* 2017, **35**(25):2885-2892.
11. Antonia SJ, Villegas A, Daniel D, Vicente D, Murakami S, Hui R, Yokoi T, Chiappori A, Lee KH, de Wit M *et al*: **Durvalumab after Chemoradiotherapy in Stage III Non-Small-Cell Lung Cancer**. *N Engl J Med* 2017, **377**(20):1919-1929.
12. Atagi S, Kawahara M, Yokoyama A, Okamoto H, Yamamoto N, Ohe Y, Sawa T, Ishikura S, Shibata T, Fukuda H *et al*: **Thoracic radiotherapy with or without daily low-dose carboplatin in elderly patients with non-small-cell lung cancer: a randomised, controlled, phase 3 trial by the Japan Clinical Oncology Group (JCOG0301)**. *The Lancet Oncology* 2012, **13**(7):671-678.

APPENDICES

Supplemental table 1 Evaluation of target lesions:

Complete Response (CR):	Disappearance of all target lesions
Partial Response (PR):	At least a 30% decrease in the sum of the longest diameter (LD) of target lesions, taking as reference the baseline sum LD.
Progressive Disease (PD):	At least a 20% increase in the sum of the LD of target lesions, taking as reference the smallest sum LD recorded since the treatment started or the appearance of one or more new lesions.
Stable Disease (SD):	Neither sufficient shrinkage to qualify for PR nor sufficient increase to qualify for PD, taking as reference the smallest sum LD since the treatment started.

Supplemental table 2 Evaluation of Non-Target Lesions

Complete Response (CR):	Disappearance of all non-target lesions and *normalization of tumor marker level.
Incomplete Response/ Stable Disease (SD):	Persistence of one or more non-target lesion(s) and/or maintenance of tumor marker level above the normal limits.
Progressive Disease (PD):	Appearance of one or more new lesions and/or unequivocal progression of existing non-target lesions.
Although a clear progression of "non-target" lesions only is exceptional, in such circumstances the opinion of the investigator will prevail.	
*Note: If tumor markers are initially above the upper normal limit, they must normalize for a patient to be considered in complete clinical response.	

Supplemental table 3 Evaluation of Best Overall Response

The best overall response is the best response recorded from the start of the treatment until disease progression/recurrence (taking as reference for progressive disease the smallest measurements recorded since the treatment started). The patient's best response assignment will depend on the achievement of both measurement and confirmation criteria.

Target lesions	Non-target lesions	New lesions	Overall response
CR	CR	No	CR

CR	Incomplete response/SD	No	PR
PR	Non-PD	No	PR
PSD	Non-PD	No	SD
PD	Any	Yes or no	PD
Any	PD	Yes or no	PD
Any	Any	Yes	PD

Supplemental table 4 ECOG performance status

Grade	Description
0	Normal activity. Fully active, able to carry on all pre-disease performance without restriction
1	Symptoms, but ambulatory. Restricted in physically strenuous activity, but ambulatory and able to carry out work of a light or sedentary nature (e.g., light housework, office work).
2	In bed <50% of the time. Ambulatory and capable of all self-care, but unable to carry out any work activities. Up and about more than 50% of waking hours.
3	In bed >50% of the time. Capable of only limited self-care, confined to bed or chair more than 50% of waking hours.
4	100% bedridden. Completely disabled. Cannot carry on any self-care. Totally confined to bed or chair.
5	Death

Common Terminology Criteria for Adverse Events V4.0 (CTCAE)

The descriptions and grading scales found in the revised NCI Common Terminology Criteria for Adverse Events (CTCAE) version 4.0 will be utilized for adverse event reporting. (http://ctep.cancer.gov/reporting/ctc.html)

Supplemental table 5：Charlson Comorbidity Index (CCI)

**Disease**	**Score**	
Myocardial infarction	0	1
Congestive heart failure (cases with exertional dyspnea, nocturnal dyspnea, or those reacting against drug therapy)	0	1
Peripheral vascular disease (including intermittent claudication, post-bypass surgery cases, gangrene, and untreated thoracoabdominal aortic aneurysm (6 cm or more))	0	1
Cerebrovascular disorder (history of cerebrovascular disorder with almost no sequelae, TIA)	0	1
Dementia	0	1
Chronic lung disease (leading to dyspnea even with mild exertion)	0	1
Connective tissue disease (SLE, polymyositis, MCTD, PMR, moderate or greater level of RA)	0	1
Peptic ulcer disease	0	1
Mild liver disease (mild liver cirrhosis without portal hypertension, chronic hepatitis)	0	1
Diabetes (excluding cases without the three major complications and those under diet therapy alone)	0	1
Hemiplegia (Includes paraplegia. Does not have to be caused by cerebrovascular disorder)	0	1
Moderate to severe renal disease (cases that are undergoing dialysis, after kidney transplant, or have uremia)	0	1
Diabetes (cases with one or more of the three major complications and with a history of hospitalization for DKA or diabetic coma)	0	1
Solid cancers other than lung cancer (cases with cancer that do not require treatment at the time of enrollment, or where lung cancer is thought to determine the prognosis)	0	1
Leukemia (acute, chronic, polycythemia vera)	0	1
Lymphoma (including lymphosarcoma, macroglobulinemia, and myeloma)	0	1
	Total	
Moderate to severe liver disorder (with portal hypertension)	0	3
	Total	
AIDS (Acquired immunodeficiency syndrome)	0	6
	Total	

Age

≦ 40 years old: 0 points

41-50 years old: 1 point

51-60 years old: 2 points

61-70 years old: 3 points

≧ 71 years old: 4 points

Age points + complications points = Total points
