# Supplementary material for: Sequential chemo-immunotherapy followed by standard versus reduced thoracic radiotherapy for older and/or frail stage III non-small-cell lung cancer: A randomized open-label cohort trial
Source: PLoS Med. 2026 May 27;23(5):e1005111. doi: 10.1371/journal.pmed.1005111 (PMC13215528; doi:10.1371/journal.pmed.1005111)
Supplement: S2 Protocol — (DOCX) [file pmed.1005111.s003.docx]

**上海交通大学医学院附属瑞金医院**

**涉及人体科研项目方案**

（适用于前瞻性研究）

| 研究名称： | 针对无法耐受同期放化疗的体弱和/或老年Ⅲ期非小细胞肺癌患者的最优放疗剂量探索-一项开放的前瞻性随机开放临床研究 |
| --- | --- |
| 方案号： |  |
| 主要研究者： | 赵胜光/项轶 |
| 所属部门： | 放射治疗科/呼吸内科 |
| 起止年限： | 2021年5月-2024年5月 |

上海交通大学医学院附属瑞金医院

二〇 21 年 08 月 11 日

版本号：V2.0

| 1. 研究摘要 | |
| --- | --- |
| 1.1 摘要 | |
| **研究名称:** | 针对无法耐受同期放化疗的体弱和/或老年Ⅲ期非小细胞肺癌患者的最优放疗剂量探索-一项开放的前瞻性干预临床研究 |
| **版本/日期** | 第2版/2021年8月11日 |
| **研究机构** | 上海交通大学医学院附属瑞金医院 |
| **主要研究组** | 赵胜光/项轶 |
| **研究类型** | 研究者发起临床研究 |
| **研究目的:** | 旨在研究序贯放化疗后免疫检查点抑制剂维持治疗在无法耐受同步放化疗的局部晚期老年和/或体弱非小细胞肺癌患者中的有效性和安全性。此外，我们旨在评估降低胸部放疗剂量在该人群中的临床疗效和安全性。 \| |
| **研究设计** | 前瞻性随机开放队列研究 |
| **首次入组标准：** | 1. 入组时年龄≥18岁；  2. 组织学确诊的不可切除的III期非小细胞肺癌；  注：对于非鳞状细胞癌：已知存在EGFR敏感突变的受试者必须排除。 \|  3. 获得患者充分知情的书面同意；  4. 经多学科团队评估认为不适合同步放化疗，原因如下之一：(1) ECOG PS评分为2；(2) 年龄≥70岁且ECOG PS 0-1；(3) 年龄≥65岁且Charlson合并症指数为1  5. 足够的骨髓、肝脏和肾脏功能  6. 预期生存期至少3个月  7. 至少有一个可测量的（RECIST 1.1标准）、可接受照射的胸部病灶 \|  8. 经组织学或细胞学证实的非小细胞肺癌 \|  9. 足够的肺功能，FEV1 >1 L 或 >预测值的30%，且DLCO >预测值的30% \| |
| **排除标准** | 1. 既往接受过针对NSCLC的化疗、免疫治疗或放疗；  2. 过去28天内接受过重大外科手术；  3. 有同种异体器官移植史、自身免疫性疾病、免疫缺陷、肝炎或HIV病史 \|  4. 未控制的并发性疾病；  5. 其他活动性恶性肿瘤  6. 软脑膜转移癌症  7. 使用免疫抑制药物  8. 妊娠或哺乳期女性 |
| **第二阶段入组标准** | 根据RECIST 1.1标准，经4-6周期化疗-免疫治疗后，治疗反应评估为完全缓解、部分缓解或疾病稳定； |
| **研究干预:** | 1. 放疗方案：符合入选标准且在化疗联合免疫结束后经评估无病情进展的患者，将在化疗方案的末次给药后六周内开始采用光子射线治疗。针对肿瘤病灶的累及野，即原发病灶及转移淋巴结，队列A采用的剂量分割为60Gy/30Fx，队列B采用的剂量分割为50Gy/25Fx。 2. 化疗方案:队列A及队列B均建议采用基于铂类的两药联合化疗方案四至六周期，如依托泊苷/长春瑞滨/紫杉醇/多西他赛/培美曲塞+铂类，其中不应包括吉西他滨。EP方案- 依托泊苷：50 mg/m²，第1-5天； 顺铂：50 mg/m²，第1, 8天； TP方案 - 紫杉醇/白蛋白紫杉醇：135–175 mg/m²，第1天；顺铂：75 mg/m²，第1天 或 卡铂：AUC=5 mg/mL/min；第1天；DP方案 多西他赛：75 mg/m²，第1天；顺铂：75 mg/m²，第1天 或 卡铂：AUC=5 mg/mL/min；第1天；AP方案 培美曲塞：500 mg/m²，第1天 顺铂：75 mg/m²，第1天 或 卡铂：AUC=5 mg/mL/min；第1天 3. 方案：队列A及队列B均应与化疗同期开始使用免疫检查点抑制剂（PD-1或者PD-L1），并在放疗结束后继续维持使用六个月或直至出现疾病进展或不可耐受的毒性反应。可接受的ICIs包括抗PD-1（帕博利珠单抗 200mg 每3周一次；纳武利尤单抗 240mg 每3周一次； 信迪利单抗 200mg 每3周一次；特瑞普利单抗 200mg 每3周一次；卡瑞利珠单抗 200mg 每3周一次；替雷利珠单抗 200mg 每3周一次）和抗PD-L1（度伐利尤单抗，1000mg 每3周一次；阿替利珠单抗 1200mg 每3周一次；舒格利单抗 1200mg 每3周一次）。 |
| **研究终点** | 主要终点：(1) 1年无进展生存期：由研究者根据实体瘤疗效评价标准（RECIST）v.1.1进行评估；  次要终点：  (1) 总生存期；  (2) 客观缓解率，由研究者根据RECIST 1.1版本进行评估 \|  (3) 治疗相关毒性发生率； \| |
| **样本量与统计分析:** | 本研究采用2阶段设计，第一阶段每个队列分别入组12例患者，1年的PFS达到16.7%，则进入第二阶段患者入组，否则将中止研究入组。考虑10%患者脱落后，各组需入组28名患者，总计56名患者。预期将在36个月时完成。患者招募预期于19个月内完成。连续数据以中位数（四分位距或范围）表示，分类数据以频数（百分比）表示。使用Kaplan-Meier法估算生存率，并使用Brookmeyer-Crowley法估算生存率的95%置信区间。 |
|  |  |

目录

0. 方案概要 ................................................................................. 1

0.1 研究设计图 .................................................................................... 1

0.2 研究目的 ................................................................................. 1

0.3 受试者 ................................................................................. 1

0.3.1 首次注册的合格标准 ....................................... 2

0.3.2 第二次注册的合格标准 .................................... 2

0.4 第二次注册的排除标准 ....................................... 2

0.5 治疗 ....................................................................................3

0.6 研究终点 ........................................................................... 3

0.7 样本量和统计分析 ................................................... 3

1. 研究背景和理论基础 .................................... 7

1.1 非小细胞肺癌的流行病学 ................................. 7

1.2 ICIs在NSCLC中的作用 ......................................................... 8

1.3 老年和/或体弱患者面临的治疗挑战........................ 8

1.4 研究理论基础 ......................................................... 9

2.0 研究目的和终点 ................................................ 9

2.1 研究目的 ..................................................................... 9

2.2 主要终点 ..................................................................... 9

2.3 次要终点 .................................................................. 9

3.0 研究设计 .............................................................................. 10

4. 患者入组和退出 ......................................................... 9

4.1 首次注册的纳入标准 .......................................... 9

4.2 主要排除标准 ............... .......................................... 10

4.3 第二阶段的纳入标准....................................... 10

4.4 退出标准 ........................................................................... 11

4.5 终止标准 ................................................................ 11

5. 放射治疗方案 ......................................................... 12

6.0 药物治疗 ........................................................................ 13

6.1 诱导化疗-免疫治疗阶段 ....................................... 14

6.2 同步放疗阶段 ................................................ 15

6.3 ICIs维持阶段 ......................................................... 16

7. ICIs的剂量调整 ................................................ 16

7.1.1 ICIs调整 ................................................................ 17

7.1.2 ICIs暂停给药标准 ................................................ 17

7.1.3 ICIs恢复给药标准 .................................... 18

7.1.4 ICIs永久停用标准 ........................... 18

7.2 化疗药物的剂量调整 ........................ 18

7.2.1 培美曲塞+卡铂/顺铂 ............................................. 18

7.2.2 紫杉醇/白蛋白紫杉醇/多西他赛/依托泊苷 + 卡铂/顺铂联合方案 .............................................................................. 19

7.2.2.1 血液学毒性 ............................................................ 19

7.2.2.2 非血液学毒性 ...................................................... 19

8. 实验室检查和肿瘤反应评估 .............................. 20

8.1 首次注册前的检查 ................................................ 20

8.2 第二次注册前的检查和评估 ........................... 20

8.2.1 患者基本信息 ......................................................... 20

8.3 诱导化疗-免疫治疗期间的评估 ......... 20

8.4 ICIs维持治疗期间的评估 ................................. 21

9. 安全性评价 ........................................................................ 21

10. 数据分析和统计方法 ............................................. 22

10.1 样本量计算 ............................................................ 22

10.2 分析集 ........................................................................ 22

10.3 缺失值的处理 ................................................... 22

10.4 统计分析的一般原则 .................................... 22

10.4.1 患者分布和脱落情况 ............................................. 22

10.4.2 人口统计学和基线特征 ................................. 23

附录 .................................................................................25

**1. 研究背景和理论基础**

| **1.1 非小细胞肺癌的流行病学** |
| --- |
| 据国家癌症中心2019年最新公布数据，肺癌位居我国恶性肿瘤发病率和死亡率的首位，年新发肺癌病例数为78.7万例，发病率为57.26/10万[[1](#_ENREF_1)]。非小细胞肺癌（non-small-cell lung cancer，NSCLC）占所有肺癌的85%。根据患者病情严重程度，非小细胞肺癌又分为I期、II期、III期和IV期。I期和II期非小细胞肺癌都处在疾病的早期阶段，临床上以治愈为目标。手术切除肺部原发病灶±术后辅助放化疗是其标准治疗方案。I期肺癌的5年生存率可以达到70%-90%，II期肺癌的5年生存率可以达到50%-70%[[2](#_ENREF_2)]。对于III期非小细胞肺癌，也被称为局部晚期肺癌，定义为肿瘤细胞虽然出现扩散但局限在胸腔内，未出现远处转移。III期非小细胞肺癌占全部肺癌的15-20%，异质性很强，大体可分为可切除和不可切除两大类。然而，大多数IIIA/B（N2）和IIIC期患者已丧失手术根治机会，对于这一部分患者，根治性同步放化疗是这一人群的标准治疗方案[[3](#_ENREF_3), [4](#_ENREF_4)]。然而接受该方案的患者中位无疾病进展生存仅为8-10个月，其5年生存率仅为15%～20%。为了提高根治性同步放化疗的疗效，既往做了很多尝试，如增加诱导化疗、增加巩固化疗、提高放疗剂量等以改善生存，然而这些传统思维下的治疗尝试，都以失败告终[[5](#_ENREF_5), [6](#_ENREF_6)]。  1.2 ICIs在NSCLC中的作用  近年来，以免疫检查点抑制剂为代表的免疫药物维持治疗的应用，极大的改善了III期不可切除非小细胞肺癌的预后[[7](#_ENREF_7), [8](#_ENREF_8)]。PACIFIC研究[[9](#_ENREF_9)]是在根治性同步放化疗基础上进行了免疫巩固治疗，对III期非小细胞肺癌无进展生存和总生存时间都有显着提高，打破了近20年来不可切除Ⅲ期NSCLC根治性同步放化疗治疗疗效的瓶颈。PACIFIC研究显示，对于局部晚期不可切除的Ⅲ期NSCLC，在接受了标准的含铂方案同步放化疗后，未发生疾病进展的患者，接受durvalumab单抗维持治疗，对比安慰剂组，2018年WCLC更新的中位PFS为17.2个月，明显优于对照组的5.6个月。2019年ASCO最新的报告显示，三年生存率高达57%。**基于该研究结果，目前NCCN指南对于III期不可切除非小细胞肺癌推荐根治性同步放化疗后未进展患者，继续予以德瓦鲁单抗免疫维持治疗。** |
| 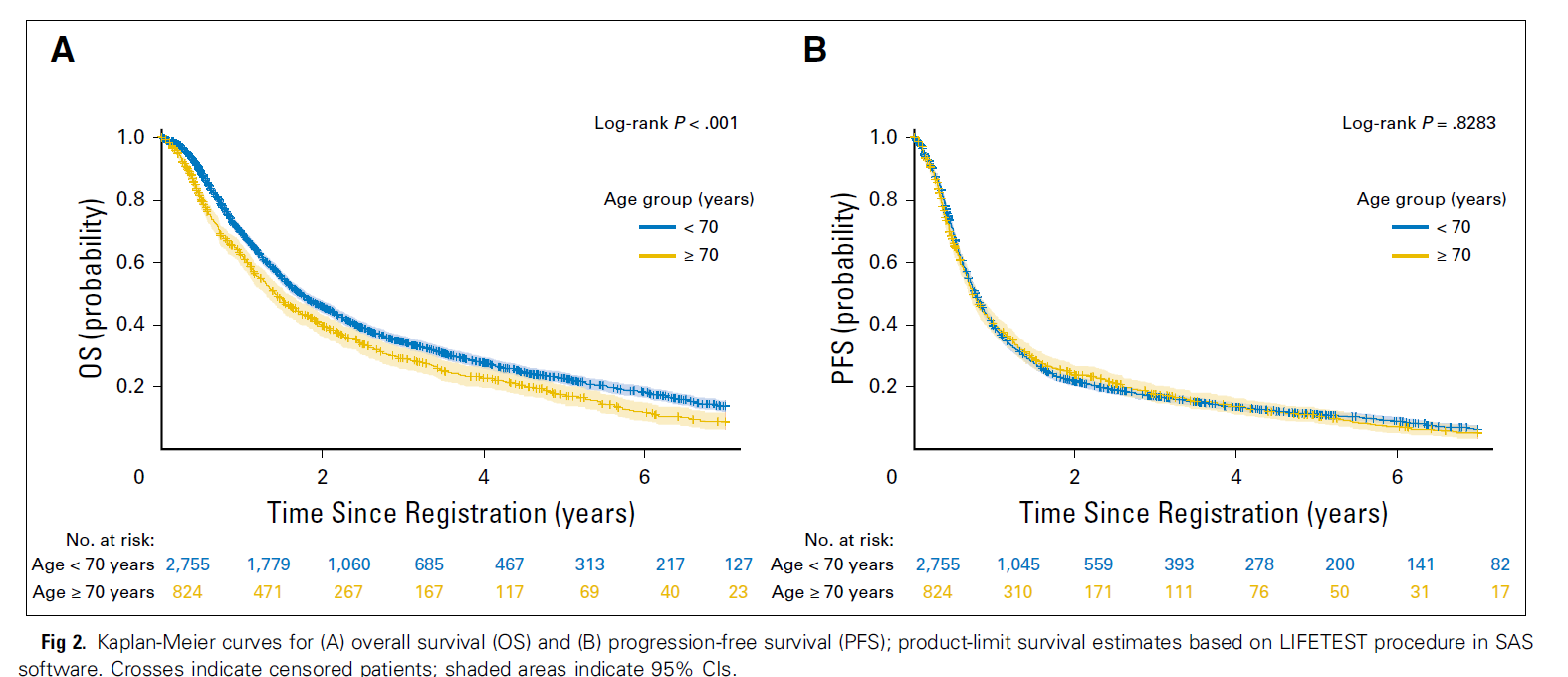1.3 老年和/体弱患者面临的治疗挑战  然而，全球许多国家都逐渐步入老龄化社会，尤以欧美、日本和我国最为显著。老龄化是一个非常严峻的社会问题，尤其给医疗界带来了巨大挑战。近年来，老年肿瘤患者数量不断增加。根据欧洲及美国的统计数据，60%以上的新发肿瘤病例及70%以上的癌症死亡均发生在65岁以上的老年人群中[[10](#_ENREF_10)]。在我国，形势也不容乐观。据中国国家癌症中心《2015中国癌症统计数据》（共统计了170万余人群的数据）显示，我国癌症发病率和死亡率均随年龄增大而逐渐增高，其中70～74岁年龄组人群的发病率为1153.65/10万，死亡率高达854.42/10万[[1](#_ENREF_1)]。**对于老年或体弱的Ⅲ期NSCLC患者，根治性同步放化疗这一治疗策略是否适用于这一人群，仍存在比较大争议。**来至美国学者Stinchcombe TE等[[11](#_ENREF_11)]回顾了1990-2012年16项临床试验的数据，分析了2768例70以下的非小细胞肺癌患者作为对照，832例70岁以上的非小细胞肺癌作为研究组，这些患者均为不能手术的局部晚期非小细胞肺癌，均采用了同步放化疗，分析两组间的生存期以及毒副反应的发生几率。研究结果显示，70岁以上组患者的生存期更差（OS：1.20,95%CI:1.09-1.31; PFS:1.17, 95%CI:1.07-1.29），并且70岁以上老年患者3级以上毒副反应明显增多（OR 1.35，95%CI:1.07-1.70），同时还观察到，70岁以上的患者同步放化疗治疗完成率比较低(47% vs. 57%, p<0.01)，中断治疗的比例高(20% vs.13%, p<0.01)，治疗期间死亡率也会增多(7.8% vs. 2.9%,p<0.01)。因此，根治性同步放化疗方案并不适用于高龄或者体弱的局部晚期非小细胞肺癌患者。 |
| **1.4 研究理论基础**  另外，PACIFIC研究亚组分析显示，有部分患者因为肿瘤负荷太大无法耐受60Gy放疗剂量，在治疗中接受了60Gy以下的放疗剂量。理论上这组患者是因肿瘤负荷大放疗剂量小，是预后较差的一组人群，但免疫治疗组无论是PFS还是OS 均一致性获益，从OS上看获益还要优于标准放疗剂量组（60-66Gy）。实际临床中，有约一半患者无法耐受60 Gy剂量放疗，如果通过联合免疫巩固治疗能够使被迫降低放疗剂量的这部分患者获益，将能使更多患者从这种治疗模式中获益。故本研究在免疫根治性序贯放疗组队列基础上，设立一组50Gy/25Fx低剂量照射老年人群队列组，欲探索免疫联合低剂量序贯放化疗的临床疗效和安全性。 |
| 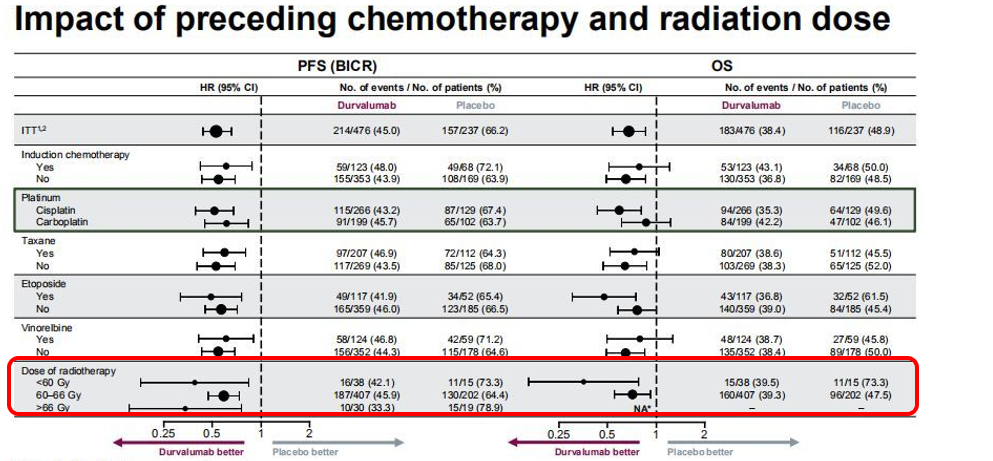  基于以上研究背景，本项目组拟开展此项前瞻性单臂双队列临床研究，探索序贯放化疗联合免疫维持治疗这一治疗模式在不能耐受同步放化疗人群的疗效和安全性，并且评估降低降低胸部放疗剂量组的临床疗效和安全性。 |
| **2.0 研究目的和终点**  **2.1 研究目的**  这是一项II期随机队列研究，旨在探讨对于未经治疗的不可切除III期NSCLC患者，采用化疗-免疫治疗后接标准或降低胸部放疗以及ICIs维持治疗的疗效和安全性。  **2.2 主要终点**  **主要终点：**接受序贯放化疗加ICIs维持治疗的两个队列的1年无进展生存率。  无进展生存期定义为从随机化到任何部位疾病复发或进展的时间。对于失访或在随访期结束时尚未死亡的患者，其数据将在最后一次可用随访时进行删失。  **2.3 次要终点**  **总生存期，**定义为从随机化到任何原因死亡的时间。对于失访或在随访期结束时尚未死亡的患者，其数据将在该患者最后一次随访时进行删失。  **客观缓解率，根据RECIST 1.1标准评估的最佳ORR。**  **治疗相关毒性发生率，**定义为两组中使用不同剂量放疗完成后3个月内，III级及以上放疗相关急性毒性（根据CTCAE 4.03）和亚急性毒性（RTOG标准）的发生率。同时评估该治疗方案的安全性，包括长期毒性（RTOG标准）。  **3.0 研究设计**  **我们的试验是一项开放的、单中心、随机、两队列的前瞻性临床研究，旨在确定对于无法耐受同步放化疗的体弱和/或老年不可切除III期NSCLC患者的最佳放疗剂量的疗效和安全性（图3）。**  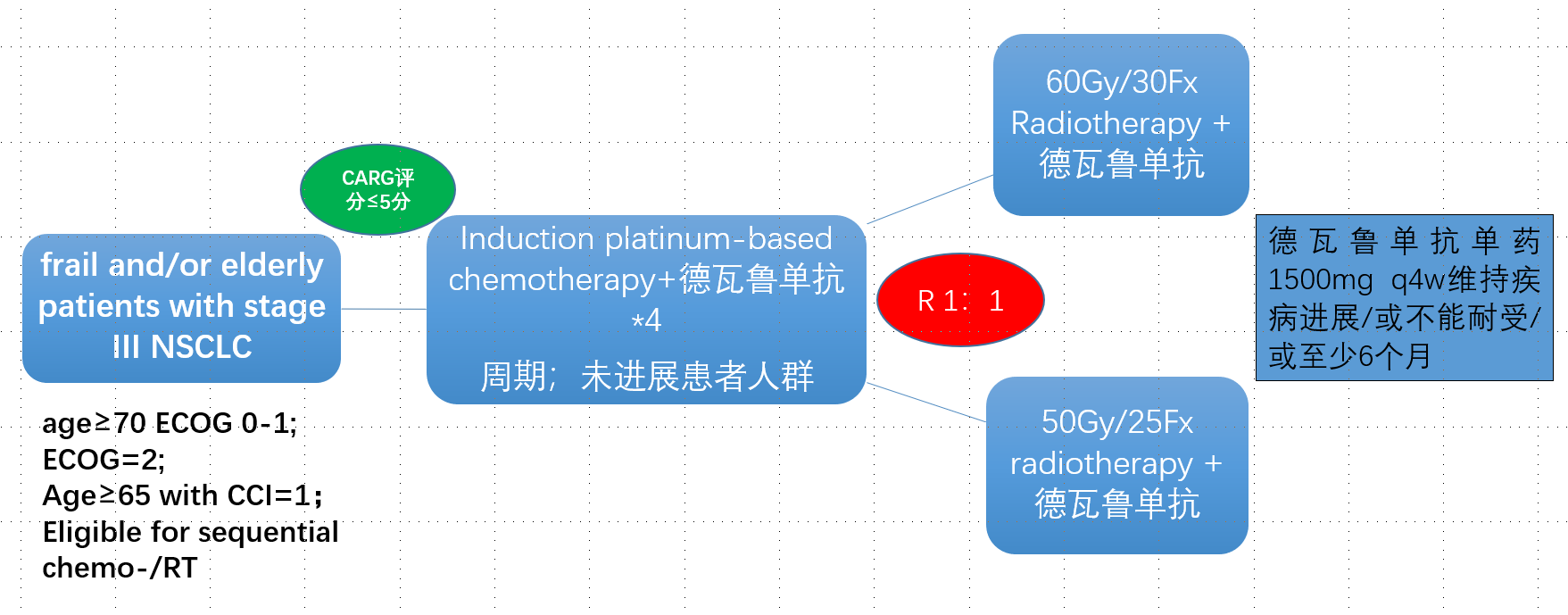 |

**图3：研究设计。CCI：查尔森合并症指数；ECOG PS：美国东部肿瘤协作组体能状态评分；ICIs：免疫检查点抑制剂；NSCLC：非小细胞肺癌；PFS：无进展生存期。**

(1) 诱导化疗-免疫治疗阶段：队列A和队列B均将接受4-6周期以铂类为基础的双药联合化疗方案联合ICIs。

(2) 同步放疗-免疫治疗阶段：放疗方案：在化疗-免疫治疗后未显示疾病进展的患者，将在最后一次系统性治疗后6周内接受光子放疗。患者将被随机分配至队列A接受60 Gy/30次放疗，队列B接受50 Gy/25次放疗；

(3) ICIs维持阶段：ICIs维持治疗至少6个月（8个周期）或直至疾病进展或出现不可接受的毒性或死亡；

**4. 患者入组和退出标准**

**4.1 首次入组的纳入标准**

1. 入组时年龄≥18岁
2. 经组织学证实的不可切除的III期NSCLC；**注：对于非鳞状细胞癌：必须排除已知存在EGFR敏感突变的受试者。**
3. 获得患者充分知情后的书面同意
4. 经多学科团队评估认为不适合同步放化疗，原因如下之一：(1) ECOG PS为2；(2) 年龄≥70岁且ECOG PS 0-1；(3) 年龄≥65岁且CCI评分为1
5. 足够的骨髓、肝和肾功能
6. 预期生存期至少3个月
7. 至少有一个可测量的（RECIST 1.1标准）、可接受照射的胸部病灶
8. 经组织学或细胞学证实的非小细胞肺癌
9. 足够的肺功能，FEV1 >1 L 或 >预计值的30%，且DLCO >预计值的30%

**4.2 主要排除标准**

1. 既往接受过针对NSCLC的化疗、免疫或放疗
2. 过去28天内接受过重大外科手术
3. 有同种异体器官移植史、自身免疫性疾病、免疫缺陷、肝炎或HIV病史
4. 未控制的合并疾病
5. 其他活动性恶性肿瘤
6. 软脑膜癌病
7. 使用免疫抑制药物
8. 妊娠或哺乳期女性

**4.3 第二阶段的纳入标准**

1. 根据RECIST 1.1标准评估，经过4-6周期化疗-免疫治疗后，治疗反应评估为完全缓解、部分缓解或疾病稳定；
2. 同意接受胸部放疗和ICIs维持治疗；

**4.4 退出标准**

受试者可以随时撤回知情同意并退出试验。在以下情况下，研究者可决定让受试者退出研究：

1. 任何可能使受试者不再获益的临床不良事件、实验室异常、妊娠事件或其他医学状况。
2. 受试者符合任何排除标准，可能无法继续参与试验（包括试验期间新出现的临床指征或未能及时发现的持续性问题）。
3. 从医学伦理角度考虑，认为有必要停止试验。
4. 依从性差的受试者，在完成所有试验前不再接受用药或检查，或在试验完成前同时接受其他抗肿瘤治疗，且无法按计划坚持完成试验。

**4.5 终止标准**

如有充分理由，本研究可能提前终止或暂停。决定方将提供说明提前终止或暂停理由的书面通知，并发送给研究者、申办方、伦理委员会和相关部门。终止本研究的原因包括但不限于以下：

1. 试验中发现临床试验方案存在重大错误，难以评估治疗结果；
2. 申办方要求终止；
3. 相关部门或伦理委员会因某种原因下令终止试验。

**5. 放射治疗方案**

所有患者在进行体位固定、定位CT、计划和治疗前，应由多学科治疗团队评估其放疗资格，并告知治疗可能的毒性和预后。符合纳入标准的患者将接受针对肿瘤病灶累及野的调强放疗/容积旋转调强放疗，包括原发灶和转移淋巴结，剂量分割为队列A 60Gy/30次，队列B 50Gy/25次。

根据射野要求设计个体化的体位固定装置，患者取仰卧位，双臂上举抱头。使用翼形板、真空垫和脚垫固定患者体位。进行胸部增强定位CT扫描，建议扫描范围从颈部至肾脏下缘，包括肿瘤和邻近重要器官，具体根据射野要求。

临床医生在定位CT的每一层上勾画大体肿瘤靶区，包括原发灶和转移淋巴结。此外，为了考虑呼吸运动，对肺癌患者使用4D CT扫描生成内大体肿瘤靶区，以涵盖整个呼吸周期中的肿瘤运动。临床靶区由GTV外扩形成，鳞癌外扩6 mm，腺癌外扩8 mm，包括有转移淋巴结的淋巴引流区，但不超出解剖边界。计划靶区定义为CTV在所有方向外扩0.5 cm。

放疗按每日一次，每周5次的计划进行。剂量优化旨在实现95%的处方剂量覆盖PTV体积，同时将正常器官的剂量保持在可耐受限度内。PTV最大剂量不允许超过处方剂量的107%，处方剂量的105% - 107%覆盖不超过PTV体积的5%。

勾画的危及器官正常组织体积包括肺、食管、脊髓和心脏。双肺平均剂量≤ 18 Gy，肺V20 ≤30%，肺V5≤65%，而食管平均剂量保持在30 Gy以下。心脏V40保持在30%以下，心脏平均剂量保持在24 Gy以下。

接受治疗的患者可能因体型变化、肿瘤退缩或副作用而需要改变计划。任何计划变更应与主治医生讨论，并及时记录原因和进一步治疗措施。应继续随访患者的毒性和疗效。

**6.0 药物治疗**

**6.1 诱导化疗-免疫治疗阶段**

**免疫检查点抑制剂：**

给药方式：在每个治疗周期的第1天，静脉输注ICIs，输注时间60分钟或以上，每3周一次（21天，Q3W）。

可接受的ICIs包括抗PD-1（帕博利珠单抗 200mg 每3周一次；**纳武利尤单抗 240mg 每3周一次；** 信迪利单抗 200mg 每3周一次；特瑞普利单抗 200mg 每3周一次；卡瑞利珠单抗 200mg 每3周一次；替雷利珠单抗 200mg 每3周一次）和抗PD-L1（度伐利尤单抗，1000mg 每3周一次；阿替利珠单抗 1200mg 每3周一次；舒格利单抗 1200mg 每3周一次）。

**化疗**

ICIs将首先给药，如果同日给予化疗，可在ICIs输注结束后30分钟开始化疗。可接受的化疗方案包括依托泊苷/长春瑞滨/紫杉醇/多西他赛/培美曲塞 + 铂类，进行4-6个周期，不包括吉西他滨。

**EP方案**

- 依托泊苷：50 mg/m²，第1-5天；
- 顺铂：50 mg/m²，第1, 8天；

**TP方案**

- 紫杉醇/白蛋白紫杉醇：135 mg/m²，第1天
- 顺铂：75 mg/m²，第1天 或 卡铂：AUC=5 mg/mL/min；第1天

**DP方案**

- 多西他赛：75 mg/m²，第1天
- 顺铂：75 mg/m²，第1天 或 卡铂：AUC=5 mg/mL/min；第1天

**AP方案**

- 培美曲塞：500 mg/m²，第1天
- 顺铂：75 mg/m²，第1天 或 卡铂：AUC=5 mg/mL/min；第1天

培美曲塞 + 铂类化疗的预处理（表1）

| **处理** | **剂量/给药途径** | **持续时间** |
| --- | --- | --- |
| 叶酸（或其他含叶酸的符合维生素产品） | 350-100 μg 口服 | 从至少Cycle1 Day1前5-7天开始每日一次给药，直至培美曲塞停用后3周 |
| 维生素B12 | 1000 μg 肌肉注射 | 在Cycle1 Day1前7天内肌肉注射一次，然后每3个周期肌肉注射一次。后续的维生素B12可以在培美曲塞给药的同一天给予。 |
| 地塞米松（或同类药物） | 4 mg 口服 | 每日两次，在每次培美曲塞输注前一天、当天和后一天。 |

肌肉注射=肌注；口服=口服。
注：预防性止吐药根据当地指南给予。

**6.2 同步放疗阶段**

体弱/老年III期NSCLC患者将被随机分配接受ICIs联合标准胸部放疗剂量60Gy/30次（标准组，队列A）或优化胸部放疗剂量50Gy/25次（优化组，队列B）。同步ICIs将在放疗期间每21天给药一次；

**6.3 ICIs维持阶段**

胸部放疗后，将继续ICIs维持治疗至少6个月（8个周期）或直至疾病进展或出现不可接受的毒性或死亡；

**7. ICIs的剂量调整**

**7.1.1 ICIs调整**

不允许对ICIs进行剂量调整。如发生任何治疗相关不良事件，应根据以下标准暂停或停用ICIs。ICIs的暂停给药不应超过12周。

**7.1.2 ICIs暂停给药标准**

任何2级药物相关不良事件（非皮肤事件），除以下情况外：

-- 对于2级药物相关疲劳或实验室检查结果异常，研究治疗无需推迟。

• 任何3级药物相关皮肤不良事件

• 任何3级药物相关实验室检查结果异常，除以下情况外：

-- 对于3级淋巴细胞减少，研究治疗无需推迟。

-- 当基线AST、ALT或总胆红素水平在正常范围内的受试者出现≥2级药物相关毒性时，研究治疗将推迟。

-- 当基线AST、ALT或总胆红素水平≤1级毒性的受试者出现≥3级药物相关毒性时，研究治疗将推迟。

**7.1.3 ICIs恢复给药标准**

当药物相关AE预计在计划给药时间（最后一次给药后12周）内9周内缓解至≤1级或基线水平时，可恢复ICIs给药，除以下情况外：

• 如果存在2级疲劳，受试者可恢复治疗。

• 如果2级皮肤毒性持续存在，但无任何3级药物相关皮肤AE的受试者可恢复治疗。

• 如果受试者基线数据显示AST/ALT或总胆红素为1级异常，尽管出现2级AST/ALT或总胆红素异常，受试者仍可恢复治疗。但如果暂停给药是由于其他原因，则对于存在2级AST/ALT或总胆红素异常的受试者恢复治疗需重新评估。

• AST/ALT和总胆红素异常符合永久停用标准（参见第7.1.4节）的受试者应永久停止研究治疗。

• 药物相关性肺毒性、腹泻或结肠炎完全恢复至基线水平后才能恢复研究治疗。如果受试者在超过1个月的皮质类固醇减量后仍持续存在1级肺炎，研究者可在与申办方医学监查员协商并获得批准后恢复其治疗。

• 对于可通过生理性激素替代疗法良好控制的药物相关内分泌疾病，研究者可在与申办方医学监查员协商并获得批准后恢复受试者的治疗。

允许因毒性中断给药三次。如果发生第四次需要中断给药的毒性，经与申办方协商后，将永久停止研究治疗。详情请参阅第7.1.4节。

**7.1.4 ICIs永久停用标准**

如果发生以下任何药物相关不良事件，将永久停用ICIs。

• 2级药物相关葡萄膜炎、眼痛或视力模糊，在恢复时间窗内尽管局部治疗或需要全身治疗仍无法恢复至1级。

• ICIs输注期间或之后发生3级或更严重的输注相关反应。

• 任何持续> 7天的3级非皮肤药物相关不良事件，除以下情况外。

-- 发生3级药物相关葡萄膜炎、肺炎、支气管痉挛、超敏反应或输注相关反应的受试者，无论不良事件持续时间长短，必须停止治疗。

-- 患有3级药物相关内分泌疾病但可通过生理性激素替代疗法良好控制的受试者无需停止研究治疗。

-- 患有3级药物相关实验室检查结果异常的受试者无需停止治疗，除以下情况外：

(1) 患有3级药物相关血小板减少症且持续> 7天或伴有出血事件的受试者应停止研究治疗。

(2) 出现以下任何肝功能检查异常的受试者应停止研究治疗：

-- AST或ALT > 5-10 x ULN 持续 > 2周

-- AST或ALT > 10 x ULN

-- 总胆红素 > 5 X ULN

-- AST或ALT > 3 X ULN 且同时总胆红素 > 2 X ULN

• 任何4级药物相关不良事件或实验室检查结果异常，除以下情况外：

-- 持续≤ 7天的4级中性粒细胞减少

-- 4级淋巴细胞减少或白细胞减少

-- 对于孤立的4级淀粉酶或脂肪酶异常，无任何胰腺炎症状或体征，当发生4级淀粉酶或脂肪酶异常时，研究者应咨询申办方医学监查员。

-- 孤立的4级电解质紊乱/异常，可在72小时内通过电解质补充/适当治疗纠正，且无任何临床后果。

-- 患有4级药物相关内分泌不良事件的受试者，若可通过生理性激素（例如皮质类固醇、甲状腺激素）替代治疗解决或良好控制（例如，肾上腺功能不全、ACTH缺乏、甲状腺功能亢进或减退或糖耐量减低），在首先与申办方医学监查员沟通并获得批准后，无需停止治疗。

• 任何导致下一次给药距离上一次给药> 12周的事件需要停止研究用药，除以下任何情况外：

**7.2 化疗药物的剂量调整**

化疗的剂量调整遵循当地临床实践和药品说明书。研究者可根据化疗单药或联合治疗后发生的不良事件的严重程度，在必要时减少剂量。如果药物相关AE已恢复至≤1级或基线水平[或对于脱发、疲劳或研究者判断不影响用药安全性的其他药物相关AE恢复至≤2级]，则允许受试者进入下一个治疗周期。允许因AE未缓解而延迟治疗周期。如果研究者判断AE由特定药物引起，并且已导致该特定药物的剂量调整或治疗延迟，只要不禁忌，可以给予另一种药物。

因药物相关AE而减少的剂量将不再增加。每种化疗药物仅允许两次减量。在第三次减量时，受试者必须暂停治疗。

如果研究者判断AE由一种化疗药物引起，则仅对该化疗药物进行剂量调整。如果AE由两种化疗药物联合引起，则对两种药物均进行剂量调整。

化疗最多可暂停6周。不良事件和实验室检查结果根据NCI CTCAE v4.03分级。所有剂量调整遵循最大剂量调整原则。剂量调整说明参见表2。

**表2 药物剂量调整**

|  | 剂量水平 0 (DL-0) | 剂量水平 1 (DL-1) | 剂量水平 2 (DL-2) | 剂量水平 3 (DL-3) |
| --- | --- | --- | --- | --- |
| 卡铂 | AUC=5，最大剂量: 750 mg | AUC 3.5，最大剂量: 562.5 mg | AUC2.5，最大剂量: 375 mg | 停药 |
| 顺铂 | 75 mg/m² | 56 mg/m² | 37.5 mg/m² | 停用 |
| 培美曲塞 | 500 mg/m² | 375 mg/m² | 250 mg/m² | 停用 |
| 紫杉醇/白蛋白紫杉醇 | 175 mg/m² | 132 mg/m² | 88 mg/m² | 停用 |
| 多西他赛 | 75 mg/m² | 56 mg/m² | 37.5 mg/m² | 停用 |
| 依托泊苷 | 50 mg/m² | 37.5 mg/m² | 25 mg/m² | 停用 |
| ICIs | 抗PD-1(帕博利珠单抗 200mg 每3周一次；纳武利尤单抗 240mg 每3周一次；信迪利单抗 200mg 每3周一次；特瑞普利单抗 200mg 每3周一次； 卡瑞利珠单抗 200mg 每3周一次； 替雷利珠单抗 200mg 每3周一次) 和抗PD-L1 (度伐利尤单抗, 1000mg 每3周一次；阿替利珠单抗 1200mg 每3周一次；舒格利单抗 1200mg 每3周一次) | 不允许 | 不允许 | 不允许 |

**7.2.1 培美曲塞+卡铂/顺铂**

培美曲塞因血液学毒性的剂量调整基于治疗周期第1天测量的中性粒细胞计数和血小板计数。下一周期的剂量将根据下一剂开始前的最低血细胞计数或非血液学毒性的最高严重程度进行调整。治疗将延迟直至患者恢复。重新开始治疗应遵循表3和表4中的剂量调整原则。实际剂量调整将由研究者根据受试者状况决定。

**表3 针对有血液学毒性的受试者，卡铂/顺铂和培美曲塞的推荐剂量调整**

|  |  | 卡铂/顺铂 | 培美曲塞 |
| --- | --- | --- | --- |
| 血小板 | 中性粒细胞计数 | 表2推荐的剂量水平 (DL) | |
| ≥50,000/mm³ | ≥500/mm³ | DL-0 | DL-0 |
| ≥50,000/mm³ | <500/mm³ | DL-1 | DL-1 |
| <50,000/mm³无出血 | 任何值 | DL-1 | DL-1 |
| <50,000/mm³有2级以上出血 | 任何值 | DL-2 | DL-2 |
| 任何值 | <1000/mm³ 且发热≥38.5℃ | DL-1 | DL-1 |

**表4 针对有非血液学毒性的受试者，卡铂/顺铂和培美曲塞的推荐剂量调整**

|  |  | 卡铂/顺铂 | 培美曲塞 |
| --- | --- | --- | --- |
| 毒性 | CTC AE grade | 表2推荐的剂量水平 (DL) | |
| 恶心或呕吐 | 3/4级 | DL-0 | DL-0 |
| 腹泻 | 3/4级 | DL-0 | DL-1 |
| 神经毒性 | 3/4级 | DL-1 | DL-1 |
| 转氨酶升高 | 3/4级 | DL-1 | DL-1 |
| 其他非血液学毒性 | 3/4级 | DL-1 | DL-1 |

**7.2.2 紫杉醇/白蛋白紫杉醇/多西他赛/依托泊苷 + 卡铂/顺铂联合方案**

**7.2.2.1 血液学毒性**

紫杉醇/白蛋白紫杉醇/多西他赛/依托泊苷因血液学毒性的剂量调整基于治疗周期第1天测量的中性粒细胞计数和血小板计数。周期开始前，ANC和血小板必须分别≥ 1500/mm³和≥100,000/mm³。下一周期的剂量将根据上一周期中最低的ANC和血小板计数进行调整。研究治疗将根据表5针对骨髓抑制进行调整或暂停。将根据当地标准和指南采取行动。推荐使用集落刺激因子而非减量来治疗发热性中性粒细胞减少或4级中性粒细胞减少。因血液学毒性暂停化疗后，每周检测一次全血细胞计数，直至计数恢复至允许治疗的最低值，之后按常规治疗方案进行。研究者可根据紫杉醇/白蛋白紫杉醇的剂量调整来调整卡铂的剂量。

表5 紫杉醇/白蛋白紫杉醇/多西他赛/依托泊苷因血液学毒性的剂量调整指南

| 血小板 | 中性粒细胞计数(ANC) | 紫杉醇/白蛋白紫杉醇/多西他赛/依托泊苷 |
| --- | --- | --- |
| ≥100,000/mm³ 且 | ≥1500/mm³ | DL-0 |
| <50,000/mm³ 或 | <500/mm³ 或发热性中性粒细胞减少 | DL-1 |
| 第二次出现 <50,000/mm³ 或 | 第二次出现 <500/mm³ 或发热性中性粒细胞减少 | DL-2 |
| 第三次出现 <50,000/mm³ 或 | 第三次出现 <500/mm³ 或发热性中性粒细胞减少 | DL-3 |

**7.2.2.2 非血液学毒性**

通常，对于严重（3级或4级）非血液学毒性和恶心/呕吐，研究者可根据判断暂停或减少紫杉醇/白蛋白紫杉醇/多西他赛/依托泊苷的剂量。对于非血液学毒性，参考表6根据毒性的严重程度和分级进行剂量调整的指南。

研究者可根据紫杉醇/白蛋白紫杉醇/多西他赛的剂量调整来调整卡铂/顺铂的剂量。对于≥3级超敏反应和≥3级神经毒性，将永久停用紫杉醇/白蛋白紫杉醇/多西他赛/依托泊苷。

表6 **非血液学毒性剂量调整指南**

|  | Grade 2 | ≥3级 |
| --- | --- | --- |
| 首次发生 | 可暂停治疗直至毒性恢复至0-1级之后如可能应继续DL-0 | 可暂停治疗直至毒性恢复至0-1级，之后如可能应继续DL-1 |
| 第二次发生相同毒性 | 可暂停治疗直至毒性恢复至0-1级之后如可能应继续DL-1 | 可暂停治疗直至毒性恢复至0-1级，之后如可能应继续DL-2 |
| 第三次发生相同毒性 | 可暂停治疗直至毒性恢复至0-1级之后如可能应继续DL-2 | 永久停止治疗 DL-3 |
| 第四次发生相同毒性 | 永久停止治疗 DL-3 | 永久停止治疗 DL-3 |

**8. 实验室检查和肿瘤反应评估**

**8.1 首次注册前的检查**

8.1.1. 应进行详细的病史采集和体格检查。

8.1.2. 需要进行原发疾病的病理诊断。

8.1.3. 应进行以下实验室检查：

(1) 外周血、尿、便常规检查，以及便潜血试验。

(2) 血液生化检查，包括肝功能（ALT、AST、ALP、黄疸指数、直接和间接胆红素、白蛋白、球蛋白、前白蛋白、凝血酶原时间、r-GT）、肾功能（肌酐、尿酸等）和心肌酶谱。

(3) 肿瘤标志物，如CEA、NSE、SCC、CA125、细胞角蛋白19片段。

(4) 梅毒抗体、HIV和全套肝炎病毒检查。

(5) 甲状腺功能检查（TSH、FT3、FT4）。

(6) 自身免疫抗体检查，包括抗Ro-52抗体、抗PM-Scl抗体、抗着丝点蛋白B抗体、抗血管核抗原抗体、抗双链DNA抗体、抗核小体抗体、抗组蛋白抗体、抗核糖体P蛋白抗体、抗M2线粒体抗体、抗RNP/Sm抗体。

(7) 应通过CD4CD8阳性细胞和CD4CD28阳性细胞评估细胞免疫功能状态。

(8) 应评估细胞因子变化，包括IL-6、IL-8、IL-10和INF-γ。

8.1.4 应进行影像学检查，包括胸部增强CT（如有增强CT禁忌症则行平扫CT）、头颅MRI、腹部超声和骨扫描，以排除远处转移。

8.1.5 应进行心功能检查，包括心电图、心脏超声、24小时动态心电图检查（针对有心脏病史的患者）和冠状动脉造影（针对有冠状动脉狭窄或心脏支架置入史的患者）。

8.1.6 需要进行肺功能测试。

**8.2 第二次注册前的检查和评估**

检查和评估将在第二次注册前进行。请注意，如果受试者在规定期限内已有检查结果，且受试者同意，则允许使用知情同意前的检查结果。获得知情同意后不必进行相同的检查。

**8.2.1 患者基本信息。**

8.2.2. 应进行以下实验室检查：

(1) 外周血、尿、便常规检查，以及便潜血试验。

(2) 血液生化检查，包括肝功能（ALT、AST、ALP、黄疸指数、直接和间接胆红素、白蛋白、球蛋白、前白蛋白、凝血酶原时间、r-GT）、肾功能（肌酐、尿酸等）和心肌酶谱。

(3) 肿瘤标志物，如CEA、NSE、SCC、CA125、细胞角蛋白19片段。

(4) 梅毒抗体、HIV和全套肝炎病毒检查。

(5) 甲状腺功能检查（TSH、FT3、FT4）。

(6) 自身免疫抗体检查，包括抗Ro-52抗体、抗PM-Scl抗体、抗着丝点蛋白B抗体、抗血管核抗原抗体、抗双链DNA抗体、抗核小体抗体、抗组蛋白抗体、抗核糖体P蛋白抗体、抗M2线粒体抗体、抗RNP/Sm抗体。

(7) 应通过CD4CD8阳性细胞和CD4CD28阳性细胞评估细胞免疫功能状态。

(8) 应评估细胞因子变化，包括IL-6、IL-8、IL-10和INF-γ。

8.2.3 应进行影像学检查，包括胸部增强CT（如有增强CT禁忌症则行平扫CT）、头颅MRI、腹部超声和骨扫描，以排除远处转移。

**8.3 诱导化疗-免疫治疗期间的评估**

在放疗期间，应至少每周评估患者对放疗的耐受性和急性毒性，并记录相关信息。每周复查血常规以观察血液学毒性，包括白细胞、中性粒细胞数、血小板和HB。每两周复查肝功能，观察患者肝脏毒性。应监测患者发热、咳嗽、咳痰、胸闷、胸痛等症状，必要时可进行肺部听诊和影像学检查以排除放射性肺炎。观察吞咽情况，评估患者食管毒性。

**8.4 ICIs维持治疗期间的评估**

每21天在ICIs给药前进行检查。每9周进行一次评估；

8.4.1. 应进行以下实验室检查：

(1) 外周血、尿、便常规检查，以及便潜血试验。

(2) 血液生化检查，包括肝功能（ALT、AST、ALP、黄疸指数、直接和间接胆红素、白蛋白、球蛋白、前白蛋白、凝血酶原时间、r-GT）、肾功能（肌酐、尿酸等）和心肌酶谱。

(3) 肿瘤标志物，如CEA、NSE、SCC、CA125、细胞角蛋白19片段。

(4) 甲状腺功能检查（TSH、FT3、FT4）。

(5) 自身免疫抗体检查，包括抗Ro-52抗体、抗PM-Scl抗体、抗着丝点蛋白B抗体、抗血管核抗原抗体、抗双链DNA抗体、抗核小体抗体、抗组蛋白抗体、抗核糖体P蛋白抗体、抗M2线粒体抗体、抗RNP/Sm抗体。

8.4.2 应进行影像学检查，包括胸部增强CT（如有增强CT禁忌症则行平扫CT）、头颅MRI、腹部超声和骨扫描，以排除远处转移。

**9. 安全性评价**

在试验期间，将根据不良事件记录、实验室检查、生命体征、体格检查、KPS评分、心脏超声和心电图记录来评估治疗方法的安全性。试验期间应密切观察受试者用药后的症状和体征。应及时有效地处理不良事件/反应，以确保受试者的安全和利益。

**10. 数据分析和统计方法**

**10.1 样本量计算**

按Simon's two-stage 统计方法设计，假设无法耐受同期放化疗的Ⅲ期NSCLC患者采用序贯放化疗维持免疫治疗后的1年无进展生存率可由20％提高至40％，队列A及队列B各预计需入组25名患者。本研究采用2阶段设计，第一阶段每个队列分别入组12例患者，1年的PFS达到16.7%，则进入第二阶段患者入组，否则将中止研究入组。考虑10%患者脱落后，各需入组28名患者，总计56名患者。


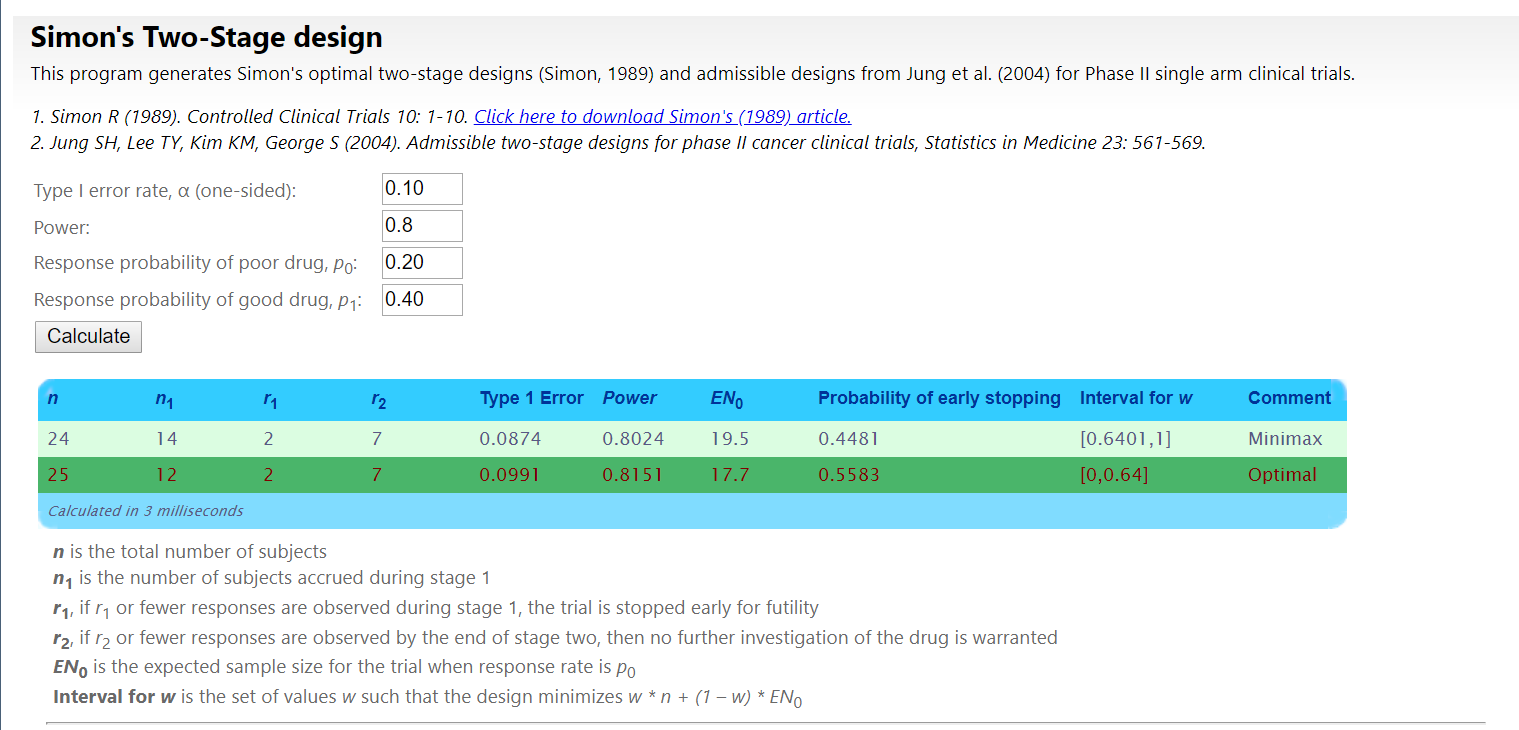


**10.2 分析集**

分析人群包括全分析集、符合方案集和安全性集。

全分析集：遵循意向性治疗原则，对所有已入组并接受治疗的病例进行疗效分析。

符合方案集：所有符合试验方案、依从性良好、至少接受过一个周期研究药物治疗（不包括入组后有充分证据证明疾病进展的患者）、试验期间未使用禁用药物并完成CRF要求的患者。不对缺失数据进行填补。疗效结果同时使用FAS和PPS进行统计分析。

安全性分析集：所有入组并接受治疗的病例，以及所有放疗或给药后有安全性记录的患者。该数据集用于安全性分析。

**10.3 缺失值的处理**

疗效指标：所有因患者提前退出而缺失的主要指标在分析中记录为“无法评估”。当计算包含时间变量（如PFS）时，通过检查停药后接受过影像学评估的受试者获得删失时间。对于基线和安全性数据，不估计缺失值。

对于因样本处理不当导致的实验室数据极端值，将在分析中使用相应的非计划访视数据，或将其视为缺失数据而不纳入分析。

**10.4 统计分析的一般原则**

除非另有说明，本研究中的数据将按照以下一般原则以描述性统计进行总结。

计量资料以均值、标准差、中位数、最大值和最小值进行总结；计数资料以频数和百分比进行总结；时间事件数据采用Kaplan-Meier法估计生存率并绘制生存曲线；血药浓度数据使用均值、标准差、变异系数、中位数、最大值和最小值进行总结。

**10.4.1 患者分布和脱落情况**

使用病例数（百分比）描述受试者的入组和完成情况。各数据集中的病例分布。列出脱落和排除患者的用药情况以及提前退出的原因。各分析集中的病例分布。

**10.4.2 人口统计学和基线特征**

对人口统计学数据和基线特征进行描述性统计。计量资料需计算病例数、均值、标准差、中位数、最小值和最大值；计数资料和等级计算频数和构成比。

**参考文献：**

1. Chen W, Zheng R, Baade PD, Zhang S, Zeng H, Bray F, Jemal A, Yu XQ, He J: **Cancer statistics in China, 2015**. *CA: a cancer journal for clinicians* 2016, **66**(2):115-132.

2. Seo YS, Kim HJ, Wu HG, Choi SM, Park S: **Lobectomy versus stereotactic ablative radiotherapy for medically operable patients with stage IA non-small cell lung cancer: A virtual randomized phase III trial stratified by age**. *Thorac Cancer* 2019, **10**(6):1489-1499.

3. Curran WJ, Jr., Paulus R, Langer CJ, Komaki R, Lee JS, Hauser S, Movsas B, Wasserman T, Rosenthal SA, Gore E *et al*: **Sequential vs. concurrent chemoradiation for stage III non-small cell lung cancer: randomized phase III trial RTOG 9410**. *Journal of the National Cancer Institute* 2011, **103**(19):1452-1460.

4. Evison M, AstraZeneca UKL: **The current treatment landscape in the UK for stage III NSCLC**. *British journal of cancer* 2020, **123**(Suppl 1):3-9.

5. Bi N, Liang J, Zhou Z, Chen D, Fu Z, Yang X, Feng Q, Hui Z, Xiao Z, Lv J *et al*: **Effect of Concurrent Chemoradiation With Celecoxib vs Concurrent Chemoradiation Alone on Survival Among Patients With Non-Small Cell Lung Cancer With and Without Cyclooxygenase 2 Genetic Variants: A Phase 2 Randomized Clinical Trial**. *JAMA network open* 2019, **2**(12):e1918070.

6. Bradley JD, Hu C, Komaki RR, Masters GA, Blumenschein GR, Schild SE, Bogart JA, Forster KM, Magliocco AM, Kavadi VS *et al*: **Long-Term Results of NRG Oncology RTOG 0617: Standard- Versus High-Dose Chemoradiotherapy With or Without Cetuximab for Unresectable Stage III Non-Small-Cell Lung Cancer**. *Journal of clinical oncology : official journal of the American Society of Clinical Oncology* 2020, **38**(7):706-714.

7. Barlesi F, Vansteenkiste J, Spigel D, Ishii H, Garassino M, de Marinis F, Ozguroglu M, Szczesna A, Polychronis A, Uslu R *et al*: **Avelumab versus docetaxel in patients with platinum-treated advanced non-small-cell lung cancer (JAVELIN Lung 200): an open-label, randomised, phase 3 study**. *The Lancet Oncology* 2018, **19**(11):1468-1479.

8. Hui R, Garon EB, Goldman JW, Leighl NB, Hellmann MD, Patnaik A, Gandhi L, Eder JP, Ahn MJ, Horn L *et al*: **Pembrolizumab as first-line therapy for patients with PD-L1-positive advanced non-small cell lung cancer: a phase 1 trial**. *Annals of oncology : official journal of the European Society for Medical Oncology / ESMO* 2017, **28**(4):874-881.

9. Antonia SJ, Villegas A, Daniel D, Vicente D, Murakami S, Hui R, Yokoi T, Chiappori A, Lee KH, de Wit M *et al*: **Durvalumab after Chemoradiotherapy in Stage III Non-Small-Cell Lung Cancer**. *The New England journal of medicine* 2017, **377**(20):1919-1929.

10. Bray F, Ferlay J, Soerjomataram I, Siegel RL, Torre LA, Jemal A: **Global cancer statistics 2018: GLOBOCAN estimates of incidence and mortality worldwide for 36 cancers in 185 countries**. *CA: a cancer journal for clinicians* 2018, **68**(6):394-424.

11. Stinchcombe TE, Zhang Y, Vokes EE, Schiller JH, Bradley JD, Kelly K, Curran WJ, Jr., Schild SE, Movsas B, Clamon G *et al*: **Pooled Analysis of Individual Patient Data on Concurrent Chemoradiotherapy for Stage III Non-Small-Cell Lung Cancer in Elderly Patients Compared With Younger Patients Who Participated in US National Cancer Institute Cooperative Group Studies**. *Journal of clinical oncology : official journal of the American Society of Clinical Oncology* 2017, **35**(25):2885-2892.

附录 I

附表1 靶病灶评估：

| 完全缓解： | 所有靶病灶消失 |
| 部分缓解： | 靶病灶最长径之和相对于基线水平减少至少30% |
| 疾病进展： | 靶病灶最长径之和相对于治疗开始后记录的最小值增加至少20%，或出现一个或多个新病灶 |
| 疾病稳定： | 既未达到PR的足够缩小，也未达到PD的足够增加，参考治疗开始后记录的最小最长径之和 |

附表2 非靶病灶评估

完全缓解： | 所有非靶病灶消失和*肿瘤标志物水平恢复正常 |
不完全缓解/ | 一个或多个非靶病灶持续存在和/或肿瘤标志物水平持续高于正常限度 |
| 疾病稳定： | |
| 疾病进展： | 出现一个或多个新病灶和/或现有非靶病灶明确进展 |
| 尽管仅"非靶"病灶明确进展的情况很罕见，在此情况下研究者的意见占主导。 |
| *注：如果肿瘤标志物最初高于正常值上限，则必须恢复正常，患者才能被视为达到完全缓解。 |

附录2 ECOG 一般状况评分

| 级别 | 说明 |
| --- | --- |
| 0 | 十分活跃, 可以不受限制地进行无疾病时的活动 |
| 1 | 不能进行重体力劳动，但能进行轻微地劳动或坐着劳动，比如轻微地家务活，办公室工作。 |
| 2 | 能走动，生活全部自理但无工作能力。能进行大约或稍多于50％的家务活。 |
| 3 | 只能对自己的生活自理，50％以上清醒的时间呆在椅子或床上。 |
| 4 | 能力完全丧失。生活完全不能自理。全部时间都得呆在床上或椅子上。 |
| 5 | 死亡 |

Am. J. Clin. Oncol. (CCT) 1982; 5:649-655

附录II 肺癌TNM分期（pTNM分期 UICC 第8版）

T分期（原发肿瘤）

pTX：未发现原发肿瘤，或者通过痰细胞学或支气管灌洗发现癌细胞，但影像学及支气管镜无法发现。

pT0：无原发肿瘤的证据。

pTis：原位癌

pT1：肿瘤最大径≤3cm，周围包绕肺组织及脏层胸膜，支气管镜见肿瘤侵及叶支气管，未侵及主支气管。

pT1mi：微小浸润性腺癌。

pT1a： 肿瘤最大径≤1cm。

pT1b： 肿瘤1cm<最大径≤2cm。

pT1c： 肿瘤2cm<最大径≤3cm。

pT2： 肿瘤3cm<最大径≤5cm；或者肿瘤侵犯主支气管（不常见的表浅扩散型肿瘤，不论体积大小，侵犯限于支气管壁时，虽可能侵犯主支气管，仍为T1），但未侵及隆突；侵及脏层胸膜；有阻塞性肺炎或者部分或全肺肺不张。符合以上任何1个条件即归为T2。

pT2a：肿瘤3cm<最大径≤4cm。

pT2b：肿瘤4cm<最大径≤5cm

pT3：肿瘤5cm<最大径≤7cm。或任何大小肿瘤直接侵犯以下任何1个器官，包括：胸壁（包含肺上沟瘤）、膈神经、心包；同一肺叶出现孤立性癌结节。符合 以上任何1个条件即归为T3。

pT4：肿瘤最大径＞7cm; 无论大小，侵及以下任何1个器官，包括：纵隔、心脏、大血管、隆突、喉返神经、主气管、食管、椎体、膈肌；同侧不同肺叶内孤立癌结节。

N-区域淋巴结

pNX：区域淋巴结无法评估。

pN0：无区域淋巴结转移。

pN1：同侧支气管周围及（或）同侧肺门淋巴结以及肺内淋巴结有转移，包括直接侵犯而累及的。

pN2：同侧纵隔内及（或）隆突下淋巴结转移。

pN3：对侧纵隔、对侧肺门、同侧或对侧前斜角肌及锁骨上淋巴结转移。

M-远处转移

MX：远处转移不能被判定。

pM1a：局限于胸腔内，对侧肺内癌结节；胸膜或心包结节；或恶性胸膜（心包）渗出液。

pM1b：超出胸腔的远处单器官单灶转移（包括单个非区域淋巴结转移）。

pM1c：超出胸腔的远处单器官多灶转移/多器官转移。

**临床分期**

隐匿性癌：TisN0M0

IA1期：T1a（mis）N0M0，T1aN0M0

IA2期：T1bN0M0

IA3期：T1cN0M0

IB期：T2aN0M0

ⅡA期：T2bN0M0

ⅡB期：T1a～cN1M0，T2aN1M0，T2bN1M0，T3N0M0

ⅢA期：T1a～cN2M0，T2a～bN2M0，T3N1M0，T4N0M0，T4N1M0

ⅢB期：T1a～cN3M0，T2a～bN3M0，T3N2M0，T4N2M0

ⅢC期：T3N3M0，T4N3M0

IVA期：任何T、任何N、M1a，任何T、任何N、M1b

IVB期：任何T、任何N、M1c

**附录3：Charlson Comorbidity Index**

将指定的疾病经适当的加权指数后加总得之，加权分数分为四种：**1分, 2分, 3分及6分**

1. **合并症评分：**

| **评分** | **疾病** | |
| --- | --- | --- |
| **1** | - 糖尿病 - 轻微的肝脏疾病 - 充血性心衰 - 心肌梗塞 - 慢性肺阻病 - 痴呆 | - 结缔组织病 - 消化性溃疡病 - 周围血管病 - 脑血管意外 - 短暂脑缺血发作 |
| **2** | - 偏瘫 - 中度至重度慢性肾病 - 任何白血病，淋巴瘤或局限性实体瘤 - 糖尿病伴器官损害 |  |
| **3** | - 中度至严重肝病 |  |
| **6** | - 转移性实体瘤 - 艾滋病 |  |

1. **年龄评分：**

- 年龄<50 ：0分
- 50-59岁：1分
- 60-69岁：2分
- 70-79岁：3分
- 年龄>80：4分
