# Supplementary material for: Sequential chemo-immunotherapy followed by standard versus reduced thoracic radiotherapy for older and/or frail stage III non-small-cell lung cancer: A randomized open-label cohort trial
Source: PLoS Med. 2026 May 27;23(5):e1005111. doi: 10.1371/journal.pmed.1005111 (PMC13215528; doi:10.1371/journal.pmed.1005111)
Supplement: S1 CONSERVE-CONSORT Checklist — (DOCX) [file pmed.1005111.s005.docx]

**CONSERVE Checklist**

| CONSERVE-CONSORT Extension: | | | | | |
| --- | --- | --- | --- | --- | --- |
| Item | Item Title | Description | | | Page No. |
| I. | Extenuating Circumstances | The trial was conducted during the COVID-19 pandemic. Between 2022 and 2024, institutional policies restricted non‑urgent clinical research, causing delays in patient screening and enrollment. | | | Section 3.1, Paragraph 1 |
| II. | Important Modifications | 1. The enrollment period was extended from the planned 12 months to 18 months (actual: September 30, 2022 – April 20, 2024). No other protocol modifications were made. | | | Section 3.1, Paragraph 1 |
|  |  | 1. Impacts and mitigating strategies: The extension ensured achievement of the target sample size (56 patients). No interim analyses were performed, and the statistical power remained unchanged. | | | Section 3.1, Paragraph 1 |
|  |  | 1. Provide a modification timeline. | | | Section 3.1, Paragraph 1 |
| III. | Responsible Parties | The principal investigator (Wei‑Xiang Qi) and the institutional review board (Ruijin Hospital, approval No.2021‑189) reviewed and approved the enrollment extension. | | | Section 2.1, Paragraph 1 |
| IV. | Interim data | No interim data were used to inform modifications; the extension was based solely on operational feasibility and safety monitoring (no early stopping rules were triggered). | | | Section 2.1, Paragraph 1 (DSMB statement) |
| CONSORT Number and Item | | For each row, if important modifications occurred check “direct impact” and/or “mitigating strategy” and describe the changes in the trial manuscript or supplement. Check “no change” for items that are unaffected in the extenuating circumstance. | | | Page No. |
|  |  | No Change | Impact* | Mitigating Strategy** |  |
| 1 | Title and abstract | X |  |  | Abstract |
| 2 | Introduction | X |  |  | Section 1.0 |
| 3 | Methods: Trial Design | X |  |  | Section 2.1 |
| 4 | Methods: Participants | X |  |  | Section 2.1 |
| 5 | Methods: Interventions | X |  |  | Section 2.5 |
| 6 | Methods: Outcomes | X |  |  | Section 2.2 |
| 7 | Methods: Sample Size | X |  |  | Section 2.6 |
| 8-10 | Methods: Randomisation | X |  |  | Section 2.5 |
| 11 | Methods: Blinding | X |  |  | NA (open-label) |
| 12 | Methods: Statistical methods |  | X | X | Section 2.6 |
| 13 | Results: Participant flow | X |  |  | Section 3.1, Figure 1 |
| 14 | Results: Recruitment | X |  |  | Section 3.1, Paragraph 1 (actual dates and reason for extension) |
| 15 | Results: Baseline data | X |  |  | Table 1 |
| 16 | Results: Numbers analysed | X |  |  | Section 3.2 |
| 17 | Results: Outcomes and estimation | X |  |  | Section 3.2, Figures 2‑3 |
| 18 | Results: Ancillary analyses | X |  |  | Section 3.2 |
| 19 | Results: Harms | X |  |  | Section 3.4, table 2 |
| 20 | Discussion: Limitations |  |  | X | Section 4.0, Paragraph 7 (acknowledges potential impact of extended enrollment on generalizability) |
| 21 | Discussion: Generalisability | X |  |  | Section 4.0 |
| 23 | Other information: Registration | X |  |  | Section 2.1, Abstract |
| 24 | Other information: Protocol | X |  |  | Supplemental 1 |
| 25 | Other information: Funding | X |  |  | Funding section |
| *Aspects of the trial that are directly affected or changed by the extenuating circumstance and are not under the control of investigators, sponsor or funder.  **Aspects of the trial that are modified by the study investigators, sponsor or funder to respond to the extenuating circumstance or manage the direct impacts on the trial. | | | | | |

Citation: Orkin AM, Gill PJ, Ghersi D, Campbell L, Sugarman J, Emsley R, Steg PG, Weijer C, Simes J, Rombey T, Williams HC, Wittes J, Moher D, Richards DP, Kasamon Y, Getz K, Hopewell S, Dickersin K, Wu T, Ayala AP, Schulz KF, Calleja S, Boutron I, Ross JS, Golub RM, Khan KM, Mulrow C, Siegfried N, Heber J, Lee N, Kearney PR, Wanyenze RK, Hróbjartsson A, Williams R, Bhandari N, Jüni P, Chan AW; CONSERVE Group. Guidelines for Reporting Trial Protocols and Completed Trials Modified Due to the COVID-19 Pandemic and Other Extenuating Circumstances: The CONSERVE 2021 Statement. JAMA. 2021.
