# Supplementary figures and images for: Sequential chemo-immunotherapy followed by standard versus reduced thoracic radiotherapy for older and/or frail stage III non-small-cell lung cancer: A randomized open-label cohort trial
Source: PLoS Med. 2026 May 27;23(5):e1005111. doi: 10.1371/journal.pmed.1005111 (PMC13215528; doi:10.1371/journal.pmed.1005111)

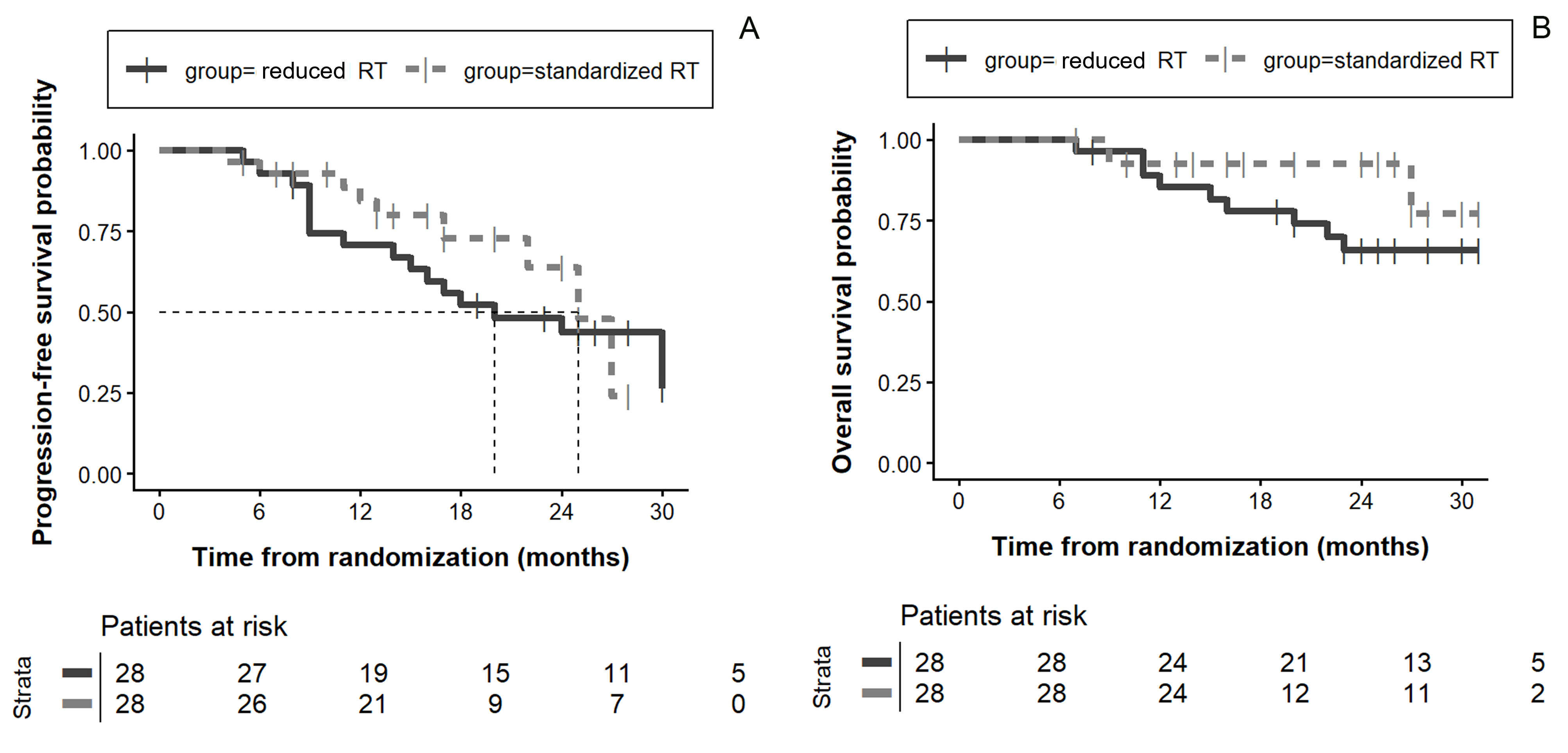

Supplement: S1 Fig — Abbreviations: RT, radiotherapy; Note: Survival curves were estimated using the Kaplan–Meier method. Between-group comparisons were descriptive only due to the non-comparative design. (TIF) [file pmed.1005111.s006.tif]
